# A double-edged sword: the role of macrophage pyroptosis-driven cytokine storm-mediated intercellular communication in tumor progression

**DOI:** 10.3389/fimmu.2026.1828486

**Published:** 2026-07-21

**Authors:** Qing-feng Wang, Hong-le Li, Hong Gao, Li-jiang Zhou, Yue Sun, Hao-ran Wang, Jia-yi Zhou

**Affiliations:** 1 Liaoning University of Traditional Chinese Medicine, Shenyang, Liaoning, China; 2 Department of Oncology, Affiliated Hospital of Liaoning University of Traditional Chinese Medicine, Shenyang, Liaoning, China

**Keywords:** cancer immunotherapy, cytokine storm, GSDMD, immunogenic cell death, inflammasome, pyroptosis, tumor microenvironment, tumor-associated macrophages

## Abstract

Pyroptosis, a lytic and inflammatory form of programmed cell death, has emerged as a regulator of tumor immunity through its capacity to trigger localized cytokine storms (operationally defined in Section 3). This review examines the dual mechanisms by which macrophage pyroptosis-driven cytokine storms influence tumor progression. Pyroptosis is executed through canonical (caspase-1/nucleotide-binding oligomerization domain–like receptor family pyrin domain–containing 3 (NLRP3)–dependent) and non-canonical (caspase-4/5/11–dependent) pathways, both converging on cleavage of gasdermin D (GSDMD) to form membrane pores that release pro-inflammatory cytokines (interleukin-1 beta, IL-1β; interleukin-18, IL-18) and damage-associated molecular patterns (DAMPs). These primary signals initiate cascade amplification through chemokine and cytokine networks, recruit diverse immune cell populations, and establish distinct inflammatory microenvironments. The effects of pyroptotic cytokine storms show striking temporal and intensity dependence. Acute, moderate inflammatory responses activate anti-tumor immunity through induction of immunogenic cell death (ICD), dendritic cell maturation, and cytotoxic lymphocyte priming. Chronic, low-grade cytokine storms, in contrast, promote tumorigenesis through six interconnected mechanisms: genomic instability and epigenetic reprogramming, cancer stem cell enrichment, pro-angiogenic remodeling, pre-metastatic niche formation, establishment of an immunosuppressive microenvironment, and induction of epithelial–mesenchymal transition. This “double-edged sword” phenomenon depends on inflammation intensity, duration, spatial distribution, and tumor microenvironment (TME) characteristics. Clinical investigations indicate that pyroptosis-related biomarkers, including GSDMD, gasdermin E (GSDME), and inflammatory cytokine profiles, may support patient stratification and treatment-response prediction across multiple cancer types in patients with tumor-associated macrophages (TAMs)–rich tumors. Preclinical evidence from bioorthogonal chemical systems in murine models, together with mathematical modeling, has suggested that pyroptosis affecting approximately 10–15% of tumor cells may serve as a tentative threshold for initiating anti-tumor immunity. However, this value has been derived from a limited number of preclinical systems (primarily 4T1 mammary tumor models) and has not yet been validated in human tumors; it should therefore be interpreted as a working hypothesis rather than an established parameter. Current therapeutic strategies targeting this pathway include NLRP3 inhibitors, IL-1β/IL-18 blockers, and combination approaches with immune checkpoint inhibitors (ICIs). Looking forward, future research should prioritize: (i) quantitative *in vivo* mapping of macrophage pyroptosis using spatial multi-omics and intravital imaging; (ii) development of tumor-targeted, spatiotemporally controlled pyroptosis inducers (e.g., nano-delivery and bioorthogonal activation systems); (iii) rational combination with immune checkpoint inhibitors and epigenetic modulators; and (iv) establishment of pyroptosis-based biomarker panels to guide patient stratification and toxicity prediction in clinical trials. Achieving “controllable cytokine storms” through precise macrophage pyroptosis modulation represents a therapeutic paradigm that balances anti-tumor efficacy against inflammatory toxicity, with the potential to advance cancer immunotherapy toward precision inflammation regulation.

## 1 Introduction

Pyroptosis is a lytic form of programmed cell death characterized by plasma membrane pore formation, cellular swelling, and the active release of pro-inflammatory mediators. Unlike apoptosis, which is “immunologically silent, “ pyroptosis generates a vigorous inflammatory signature that actively reshapes the surrounding tissue (1). This distinction is increasingly recognized as central to tumor immunology, because the immunogenic nature of pyroptosis positions it at the interface between innate immune activation and anti-tumor adaptive responses.

At the molecular level, pyroptosis is executed by the gasdermin protein family, predominantly gasdermin D (GSDMD) and gasdermin E (GSDME). Two activation routes converge on GSDMD cleavage: the caspase-1/inflammasome-dependent pathway and the caspase-4/5/11–dependent (non-canonical) pathway that senses cytosolic lipopolysaccharide (LPS) (2, 3). Cleaved GSDMD oligomerizes on the plasma membrane to form pores that release mature interleukin-1 beta (IL-1β) and interleukin-18 (IL-18). In parallel, chemotherapy-activated caspase-3 can cleave GSDME, providing an additional executioner route particularly relevant to tumor cells (4). Detailed biochemical signatures of pyroptosis are discussed in Section 2.

Within solid tumors, tumor-associated macrophages (TAMs) constitute one of the most abundant immune populations and a major source of inflammasome-driven cytokines (5). The traditional M1/M2 dichotomy, while conceptually useful, inadequately captures TAM heterogeneity. A recent synthesis of macrophage polarization, integrating the molecular logic of M1/M2 axes with newer single-cell and spatial data, has reinforced this view and catalogued the principal druggable nodes along the polarization axis (6). Recent single-cell RNA sequencing studies reveal a continuum of TAM states defined less by classical polarization than by Toll-like receptor (TLR) priming, interferon signaling history, DAMP exposure, and phagocytic burden (7–9). TAMs orchestrate intercellular communication through cytokine secretion, exosome trafficking, and direct cell–cell contact (10), placing them as central “hubs” where pyroptotic signals are generated, amplified, and propagated. Beyond their function as cytokine producers, TAMs have also been framed as double-edged regulators of immunotherapy response, acting both as effectors and as barriers of immune checkpoint blockade (11); the present review focuses on a narrower question, namely how pyroptotic execution within the TAM compartment generates the localized inflammatory signal that conditions the surrounding microenvironment.

The tumor microenvironment (TME) actively conditions macrophage pyroptosis through metabolic, immunosuppressive, and structural mechanisms. Tumor-derived lactate, hypoxia-driven hypoxia-inducible factor 1-alpha (HIF-1α) signaling, transforming growth factor beta (TGF-β), programmed death-ligand 1 (PD-L1), prostaglandin E2 (PGE2), and extracellular matrix stiffness have each been proposed to elevate the pyroptotic activation threshold, thereby contributing to immune evasion (12, 13). Therapeutic interventions, including chemotherapy, radiotherapy, and targeted agents, have been reported to overcome these restraints and restore pyroptotic competence in TAMs (14).

The term “cytokine storm” has traditionally denoted systemic hyperinflammatory syndromes such as chimeric antigen receptor T-cell (CAR-T)–associated cytokine release syndrome (CRS), sepsis, and severe viral infection (15). In the present review the more restricted operational term “localized micro-cytokine storm” is used to describe spatially confined, transient, high-amplitude cytokine release within TME inflammatory hotspots; this usage is adopted for descriptive economy and is not intended to imply equivalence with systemic CRS. The novelty of this review lies in integrating three elements that have typically been treated separately: (i) macrophage-intrinsic pyroptotic mechanisms, (ii) localized cascade amplification dynamics within the TME, and (iii) temporally and spatially resolved “double-edged sword” outcomes of pyroptosis-driven inflammation. We further propose an explicit acute/localized versus chronic/sustained framework as the principal determinant of opposing tumor outcomes, which to our knowledge has not been systematically articulated in prior reviews. Accordingly, the present review aims to: (a) summarize the molecular execution of macrophage pyroptosis and its biochemical signatures; (b) dissect how pyroptosis-released mediators propagate into localized cytokine cascades within the TME; (c) delineate the acute-versus-chronic dichotomy governing anti-tumor and pro-tumor outcomes; and (d) outline clinical and translational implications, including biomarkers, current therapeutic agents, and emerging combination strategies.

### 1.1 Literature search and review scope

This article is a narrative, integrative review and does not follow PRISMA criteria for systematic reviews or meta-analyses. PubMed, Web of Science Core Collection, and Scopus were searched from inception to October 2025 using combinations of the following terms: (“pyroptosis” OR “gasdermin” OR “GSDMD” OR “GSDME” OR “inflammasome” OR “NLRP3”) AND (“macrophage” OR “tumor-associated macrophage” OR “TAM”) AND (“cytokine storm” OR “cytokine release syndrome” OR “tumor microenvironment” OR “cancer immunotherapy”). Only English-language articles were considered. Inclusion focused on original mechanistic studies, single-cell and spatial transcriptomic analyses, structural and biochemical work on the gasdermin family, clinical and translational studies on pyroptosis-related biomarkers and inhibitors, and prior reviews providing conceptual framing. Studies were excluded if pyroptosis was not directly examined or if the data did not concern macrophages, tumor cells, or the TME. Hand-searches of the reference lists of seminal reviews and clinical trials were used to supplement the database queries. Because formal risk-of-bias assessment and quantitative synthesis were not undertaken, the present synthesis should be interpreted as an integrative discussion of converging evidence rather than as a graded evaluation of effect estimates.

Pyroptosis is a lytic form of programmed cell death characterized by plasma membrane pore formation, cellular swelling, and the active release of pro-inflammatory mediators. Unlike apoptosis, which is “immunologically silent, “ pyroptosis generates a vigorous inflammatory signature that actively reshapes the surrounding tissue (1).This distinction is increasingly recognized as central to tumor immunology, because the immunogenic nature of pyroptosis positions it at the interface between innate immune activation and anti-tumor adaptive responses.

At the molecular level, pyroptosis is executed by the gasdermin protein family, predominantly gasdermin D (GSDMD) and gasdermin E (GSDME). Two activation routes converge on GSDMD cleavage: the caspase-1/inflammasome-dependent pathway and the caspase-4/5/11-dependent (non-canonical) pathway that senses cytosolic lipopolysaccharide (LPS) (2, 3). Cleaved GSDMD oligomerizes on the plasma membrane to form pores that release mature interleukin-1β (IL-1β) and IL-18. In parallel, chemotherapy-activated caspase-3 can cleave GSDME, providing an additional executioner route particularly relevant to tumor cells (4). Detailed biochemical signatures of pyroptosis are discussed in Section 2.

Within solid tumors, tumor-associated macrophages (TAMs) constitute one of the most abundant immune populations and a major source of inflammasome-driven cytokines (5). The traditional M1/M2 dichotomy, while conceptually useful, inadequately captures TAM heterogeneity. Recent single-cell RNA sequencing studies reveal a continuum of TAM states defined less by classical polarization than by TLR priming, interferon signaling history, DAMP exposure, and phagocytic burden (7–9). TAMs orchestrate intercellular communication through cytokine secretion, exosome trafficking, and direct cell–cell contact (10), placing them as central “hubs” where pyroptotic signals are generated, amplified, and propagated.

The tumor microenvironment (TME) actively conditions macrophage pyroptosis through metabolic, immunosuppressive, and structural mechanisms. Tumor-derived lactate, hypoxia-driven HIF-1α signaling, TGF-β, PD-L1, PGE2, and extracellular matrix stiffness have each been proposed to elevate the pyroptotic activation threshold, thereby contributing to immune evasion (12, 13). Conversely, certain therapeutic interventions, including chemotherapy, radiotherapy, and targeted agents, have been reported to overcome these restraints and restore pyroptotic competence in TAMs (14).

The term “cytokine storm” has traditionally denoted systemic hyperinflammatory syndromes such as CAR-T-associated cytokine release syndrome (CRS), sepsis, and severe viral infection (15). In this review we use the more restricted operational term “localized micro-cytokine storm” to describe spatially confined, transient, high-amplitude cytokine release within TME inflammatory hotspots; this usage is adopted for descriptive economy and is not intended to imply equivalence with systemic CRS. The novelty of this review lies in integrating three elements that have typically been treated separately: (i) macrophage-intrinsic pyroptotic mechanisms, (ii) localized cascade amplification dynamics within the TME, and (iii) temporally and spatially resolved “double-edged sword” outcomes of pyroptosis-driven inflammation. We further propose an explicit acute/localized versus chronic/sustained framework as the principal determinant of opposing tumor outcomes, which to our knowledge has not been systematically articulated in prior reviews. Accordingly, the present review aims to: (a) summarize the molecular execution of macrophage pyroptosis and its biochemical signatures; (b) dissect how pyroptosis-released mediators propagate into localized cytokine cascades within the TME; (c) delineate the acute-versus-chronic dichotomy governing anti-tumor and pro-tumor outcomes; and (d) outline clinical and translational implications, including biomarkers, current therapeutic agents, and emerging combination strategies.

## 2 Molecular mechanisms of macrophage pyroptosis

Cell-type scope of this review. The primary focus of this review is macrophage (TAM) pyroptosis and its downstream consequences. Tumor-cell–intrinsic pyroptosis, especially GSDME-mediated, chemotherapy-induced pyroptosis (4, 16), is discussed in parallel because it directly shapes the DAMP and cytokine milieu sensed by TAMs, and because the two compartments are coupled *in vivo*. **Table 1** maps the principal pyroptotic events covered in Sections 2–4 by cellular compartment, indicating in each case whether the event is dominantly TAM-intrinsic, tumor-cell–intrinsic, or bidirectional.

**Table 1:** Major pyroptotic events and pathways referenced in this review, classified by cellular compartment, representative trigger, key executioner, and section cross-reference.

Event/pathway	Cellular compartment	Representative trigger	Key executioner	Section
NLRP3 inflammasome – caspase-1 – GSDMD	TAM (dominant)	PAMPs/DAMPs with ATP or K^+^ efflux	GSDMD-NT	2.1.1, 2.2
Non-canonical caspase-4/5/11 – GSDMD	TAM (dominant)	Cytosolic LPS	GSDMD-NT	2.1.1
GSDME cleavage by caspase-3	Tumor cell (dominant)	Chemotherapy (anthracyclines, cisplatin)	GSDME-NT	2.1.2
GSDMB cleavage by granzyme A	Tumor cell (CTL/NK-induced)	Cytotoxic lymphocyte attack	GSDMB-NT	2.1.2
HMGB1 release as ICD signal	Tumor cell (primary); TAM (secondary)	Pyroptosis, necroptosis, chemotherapy	HMGB1 (oxidized forms)	3.1.2, 4.2.2
Calreticulin exposure (“eat-me” signal)	Tumor cell	ICD-inducing therapies	CRT (surface translocation)	4.2.2
IL-1β/IL-18 processing and release	TAM (dominant source in TME)	Inflammasome activation	Caspase-1	3.1.1
Cytokine cascade amplification (IL-6, TNF-α, CXCL8)	TAM and stromal cells	IL-1β and DAMPs	NF-κB/STAT3	3.1.3
Bystander pyroptosis in tumor cells	Tumor cell (secondary)	High extracellular ATP, IFN-γ	GSDMD or GSDME	3.2.1

The cellular compartment column indicates the dominant source of each event in the tumor microenvironment; events labelled “primary/secondary” or “dominant/induced” reflect quantitative rather than absolute distinctions, and minor contributions from other compartments are discussed in the corresponding section.

ATP, adenosine triphosphate; CRT, calreticulin; CTL, cytotoxic T lymphocyte; CXCL8, CXC chemokine ligand 8 (IL-8); DAMP, damage-associated molecular pattern; GSDMB, gasdermin B; GSDMB-NT, gasdermin B N-terminal fragment; GSDMD, gasdermin D; GSDMD-NT, gasdermin D N-terminal fragment; GSDME, gasdermin E (DFNA5); GSDME-NT, gasdermin E N-terminal fragment; HMGB1, high mobility group box 1; ICD, immunogenic cell death; IFN-γ, interferon gamma; IL-1β, interleukin-1 beta; IL-6, interleukin-6; IL-18, interleukin-18; K^+^, potassium ion; LPS, lipopolysaccharide; NF-κB, nuclear factor kappa B; NK, natural killer (cell); NLRP3, NLR family pyrin domain containing 3; PAMP, pathogen-associated molecular pattern; STAT3, signal transducer and activator of transcription 3; TAM, tumor-associated macrophage; TME, tumor microenvironment; TNF-α, tumor necrosis factor alpha.

### 2.1 Canonical and Non-canonical pathways of pyroptosis

#### 2.1.1 Caspase-1/4/5/11–dependent pyroptotic pathways

Pyroptosis can be initiated through two principal routes, canonical (caspase-1/inflammasome-dependent) and non-canonical (caspase-4/5/11-dependent), both of which converge on gasdermin cleavage as the executioner step (2). Both routes liberate the pore-forming GSDMD N-terminal fragment (GSDMD-NT), leading to mature IL-1β and IL-18 release and lytic cell death. Membrane integrity is dynamically arbitrated by endosomal sorting complex required for transport III (ESCRT-III)–mediated repair and ninjurin-1 (NINJ1)–dependent rupture (further discussed in Section 2.1.2) (17, 18).

**Figure 1:**
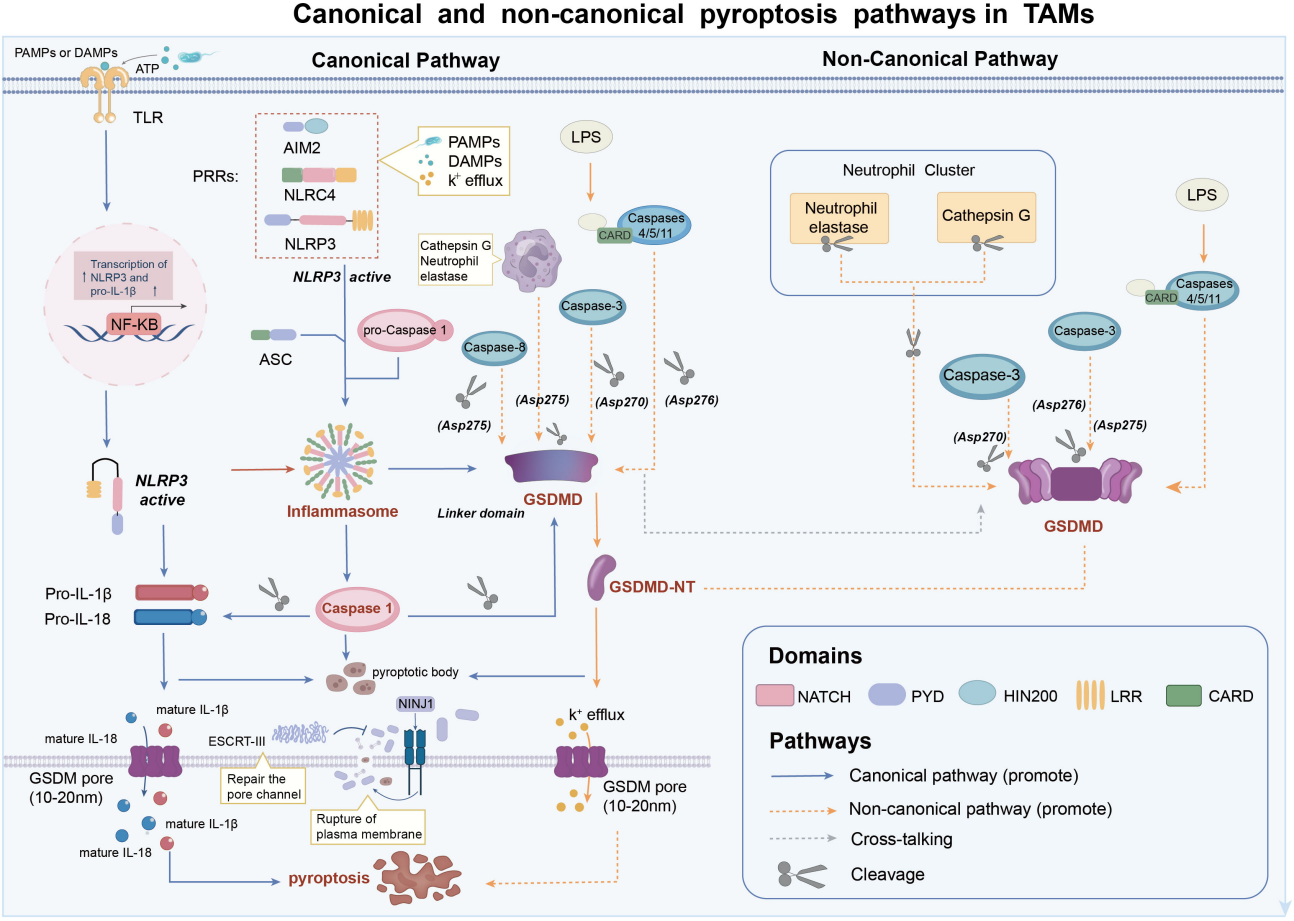
Canonical and non-canonical pathways of pyroptosis in tumor-associated macrophages (TAMs). The schematic summarises two convergent pyroptotic routes. Left (canonical pathway, blue solid arrows): recognition of pathogen- or damage-associated molecular patterns (PAMPs/DAMPs) by Toll-like receptors (TLRs) licenses NF-κB-dependent transcription of NLRP3 and pro-IL-1β; assembly of the NLRP3 (or AIM2/NLRC4) inflammasome via apoptosis-associated speck-like protein containing a CARD (ASC) drives caspase-1 activation, which cleaves GSDMD at the conserved Asp275 linker site and processes pro-IL-1β/pro-IL-18 into their mature forms. Right (non-canonical pathway, orange dashed arrows): cytosolic LPS is recognised directly by caspase-4/5 (human) or caspase-11 (mouse) via their CARD domain, leading to GSDMD cleavage and consequent K^+^ efflux that secondarily activates the NLRP3 canonical inflammasome. Convergence (centre): GSDMD-NT oligomerises on the plasma membrane to form 10–20 nm pores permitting selective IL-1β/IL-18 release; ESCRT-III mediates transient sub-lytic membrane repair, whereas NINJ1 promotes terminal rupture and pyroptotic body release. Alternative cleavage routes by caspase-8 (Asp275), caspase-3 (Asp270), and neutrophil-derived cathepsin G/elastase (Asp276) are indicated. Colour code: blue solid = canonical pathway; orange dashed = non-canonical pathway; gray dashed = cross-talk; scissors = proteolytic cleavage. Domain key: NACHT, PYD, HIN200, LRR, CARD. AIM2, absent in melanoma 2; ASC, apoptosis-associated speck-like protein containing a CARD; CARD, caspase activation and recruitment domain; DAMPs, damage-associated molecular patterns; ESCRT-III, endosomal sorting complexes required for transport III; GSDMD, gasdermin D; GSDMD-NT, GSDMD N-terminal fragment; HIN200, hematopoietic interferon-inducible nuclear protein with a 200 amino acid repeat; IL, interleukin; LPS, lipopolysaccharide; LRR, leucine-rich repeat; NACHT, NAIP–CIITA–HET-E–TP1 domain; NF-κB, nuclear factor κB; NINJ1, ninjurin-1; NLRC4, NLR family CARD domain–containing 4; NLRP3, NLR family pyrin domain–containing 3; PAMPs, pathogen-associated molecular patterns; PRRs, pattern recognition receptors; PYD, pyrin domain; TLR, Toll-like receptor. the original wording “NATCH” in the Domain key was a typographical slip; the correct domain abbreviation is NACHT (NAIP–CIITA–HET-E–TP1). HIN200, LRR, and PYD have been added to the abbreviation list since they appear in the Domain key.

In the canonical pathway, inflammasome formation is triggered by macrophage recognition of pathogen-associated molecular patterns (PAMPs) or DAMPs through pattern recognition receptors (PRRs) and assembles around sensor proteins such as NLRP3, NLR family CARD domain–containing 4 (NLRC4), absent in melanoma 2 (AIM2), and Pyrin, with NLRP3 being the most extensively characterized (see canonical arm of **Figure 1**) (19). NLRP3 activation typically follows a two-step “priming + activation” model: priming via Toll-like receptor (TLR) or cytokine receptor engagement upregulates NLRP3 and pro-IL-1β through nuclear factor kappa B (NF-κB), while activation is delivered by ATP, K^+^ efflux, reactive oxygen species (ROS), lysosomal damage, or mitochondrial dysfunction with cytosolic release of oxidized mitochondrial DNA (20, 21). Apoptosis-associated speck-like protein containing a CARD (ASC)–mediated recruitment then activates caspase-1, which cleaves GSDMD to release GSDMD-NT and processes pro-IL-1β/pro-IL-18 into their mature forms (22, 23). Detailed structural information on GSDMD pore architecture and oligomeric stoichiometry is provided in dedicated cryo-electron microscopy studies (23).

The non-canonical pathway is executed by caspase-4/5 (human) or caspase-11 (mouse), which directly recognize cytosolic LPS via their caspase recruitment domain (CARD) without upstream inflammasome assembly (24, 25) (see non-canonical arm of **Figure 1**). Although these caspases cannot directly process pro-IL-1β/IL-18, GSDMD pore formation triggers K^+^ efflux that secondarily activates the NLRP3 canonical inflammasome, restoring cytokine maturation and providing a built-in “two-step” safety mechanism for gram-negative bacterial defense (26).

Alternative GSDMD-cleaving proteases broaden this regulatory landscape. Caspase-8 cleaves GSDMD when caspase-1 activity is unavailable, for example during *Yersinia* infection or in selected tumor contexts (27, 28). Neutrophil-derived serine proteases (neutrophil elastase, cathepsin G) generate a GSDMD-dependent lytic program associated with neutrophil extracellular trap formation (NETosis) (29). These observations indicate that pyroptotic execution is not strictly confined to the caspase-1/4/5/11 axis.

#### 2.1.2 Roles of the GSDMD/GSDME protein family

The gasdermin family comprises six members (GSDMA, GSDMB, GSDMC, GSDMD, GSDME/DFNA5, and DFNB59), all sharing a conserved architecture in which an N-terminal pore-forming domain is held in check by an autoinhibitory C-terminal domain until proteolytic cleavage at the inter-domain linker (26) (see cleavage sites in **Figure 1**). Within tumor immunology, two members carry the principal weight: GSDMD (predominantly in TAMs, downstream of caspase-1/4/5/11) and GSDME (predominantly in tumor cells, downstream of caspase-3 activated by chemotherapy or other stress signals).

Cell-type scope: tumor-cell-intrinsic; included here because tumor-cell pyroptosis directly shapes the DAMP/cytokine milieu sensed by TAMs.

GSDMD is the convergence point of canonical and non-canonical pyroptosis. It is cleaved by caspase-1 and caspase-4/5/11; under conditions of caspase-1 unavailability, caspase-8 provides a backup (25, 27, 28); neutrophil serine proteases generate an alternative cleavage product relevant to NETosis (29). Following cleavage, GSDMD-NT engages plasma membrane phosphoinositides and cardiolipin to form pores that permit IL-1β and IL-18 release (22, 23). Pore-mediated cell rupture is further regulated by NINJ1, which promotes terminal plasma membrane disruption (17), whereas the ESCRT-III complex repairs sub-lytic GSDMD damage by vesiculation and thereby restrains pyroptotic commitment when activation is mild (18) (see green and red elements at the bottom of **Figure 1**).

GSDME is silenced by promoter methylation in many tumors, a pattern that has been proposed to enable escape from chemotherapy-induced pyroptosis (30). When GSDME is re-expressed, caspase-3 activated by chemotherapeutic agents (e.g., doxorubicin, etoposide, cisplatin) cleaves GSDME to generate the pore-forming GSDME-NT, converting an otherwise apoptotic stimulus into an immunogenic pyroptotic event (4, 16). Restoration of GSDME has accordingly been reported to enhance chemotherapeutic anti-tumor effects and to improve responses to immune checkpoint inhibitors (4). An extended discussion of the multifaceted oncological roles of GSDME, including its tumor-suppressor function, its silencing by promoter hypermethylation in many epithelial cancers, and the persisting technical obstacles in restoring its expression for therapeutic benefit, has been provided elsewhere (31); the present treatment retains a narrower scope and discusses GSDME mainly as an upstream determinant of the DAMP and cytokine milieu sensed by TAMs, rather than as a stand-alone therapeutic target.

Cell-type scope: tumor-cell-intrinsic; triggered by CTL/NK attack rather than intrinsic inflammasome activation.

A third tumor-relevant axis is GSDMB: cytotoxic T lymphocyte (CTL)–derived granzyme A cleaves GSDMB in target tumor cells, providing a CTL/natural killer (NK)–driven pyroptotic effector arm distinct from caspase- or chemotherapy-driven routes (32). These observations indicate the functional diversity of the gasdermin family across cellular compartments and provide multiple entry points for therapeutic intervention.

### 2.2 Specificity of macrophage pyroptosis

#### 2.2.1 Determinants of pyroptosis susceptibility in tumor-associated macrophages

The M1/M2 polarization axis has historically been used as a proxy for macrophage pyroptotic sensitivity, with M1 macrophages regarded as “primed” and M2 macrophages as “resistant.” While this dichotomy retains pedagogical value, single-cell transcriptomic analyses of human tumors now show that TAMs exist along a multidimensional continuum, with at least 6–10 transcriptionally distinct states (e.g., SPP1^+^, C1Q^+^, FCN1^+^, TREM2^+^, interferon-stimulated gene–positive (ISG^+^), and MKI67^+^ subsets) that do not map cleanly onto M1/M2 categories (7, 9, 33, 34). Pyroptosis susceptibility is therefore better understood as the integrated output of four partially independent regulatory inputs.

##### 2.2.1.1 TLR priming status

Recent engagement of TLR4 (by LPS or endogenous ligands such as high-mobility group box 1 (HMGB1), heat shock proteins (HSPs), S100 proteins) or TLR2/TLR7/TLR9 licenses macrophages for inflammasome activation by transcriptionally upregulating NLRP3, pro-IL-1β, and pro-IL-18 (35, 36). Unprimed macrophages, regardless of polarization state, exhibit low basal inflammasome readiness.

##### 2.2.1.2 Interferon signaling history

Type I IFNs (IFN-α/β, via cyclic GMP–AMP synthase–stimulator of interferon genes (cGAS–STING) or TLR–TRIF) and type II IFN (IFN-γ) both enhance pyroptotic competence: type I IFN by upregulating caspase-11 and guanylate-binding proteins (GBPs) relevant to non-canonical pyroptosis, and type II IFN by reinforcing NLRP3 priming and GSDMD expression. Tumor regions with active IFN signaling thus tend to harbor more pyroptosis-competent TAMs, independent of classical polarization markers.

##### 2.2.1.3 DAMP and metabolite exposure

Cumulative exposure to ATP, oxidized mitochondrial DNA (ox-mtDNA), urate crystals, lactate, and hypoxia shapes the activation threshold. Lactate, abundant in tumors, engages G protein–coupled receptor 81 (GPR81) and induces histone lactylation, which has been reported to both dampen and, in other contexts, rewire inflammasome output (12). Hypoxia stabilizes HIF-1α with context-dependent pro- or anti-inflammatory consequences (13).

##### 2.2.1.4 Phagocytic burden and efferocytosis history

Macrophages engaged in sustained uptake of apoptotic debris (efferocytosis) acquire an anti-inflammatory, TGF-β/interleukin-10 (IL-10)–producing state with dampened inflammasome output, independent of M1/M2 classification (37, 38). In tumors with high spontaneous apoptosis (e.g., central necrotic regions), surrounding TAMs may therefore be paradoxically pyroptosis-resistant.

##### 2.2.1.5 Residual role of metabolism

Metabolic state modulates, but does not determine, the above inputs. Glycolytic reprogramming, succinate accumulation, and HIF-1α stabilization lower the NLRP3 activation threshold (39), whereas oxidative phosphorylation (OXPHOS)–dominant metabolism is permissive but not protective. Mitochondrial dysfunction with cytosolic mtDNA release acts as a key upstream NLRP3 trigger (21). Metabolism is therefore best viewed as one input among several rather than as a master regulator.

Subset-level heterogeneity further illustrates the inadequacy of the M1/M2 framework. SPP1^+^ TAMs, despite displaying some M2-like markers, retain pyroptosis competence under appropriate stimulation (34). ISG^+^ TAMs (high interferon-stimulated gene expression) are strongly primed for both canonical and non-canonical pyroptosis. TREM2^+^ TAMs, associated with lipid-rich and immunosuppressive states, show reduced pyroptotic output even when NLRP3 is present. These observations suggest that therapeutic strategies aiming to induce TAM pyroptosis may benefit from being informed by single-cell subset profiling rather than by bulk M1/M2 ratios.

**Figure 2:**
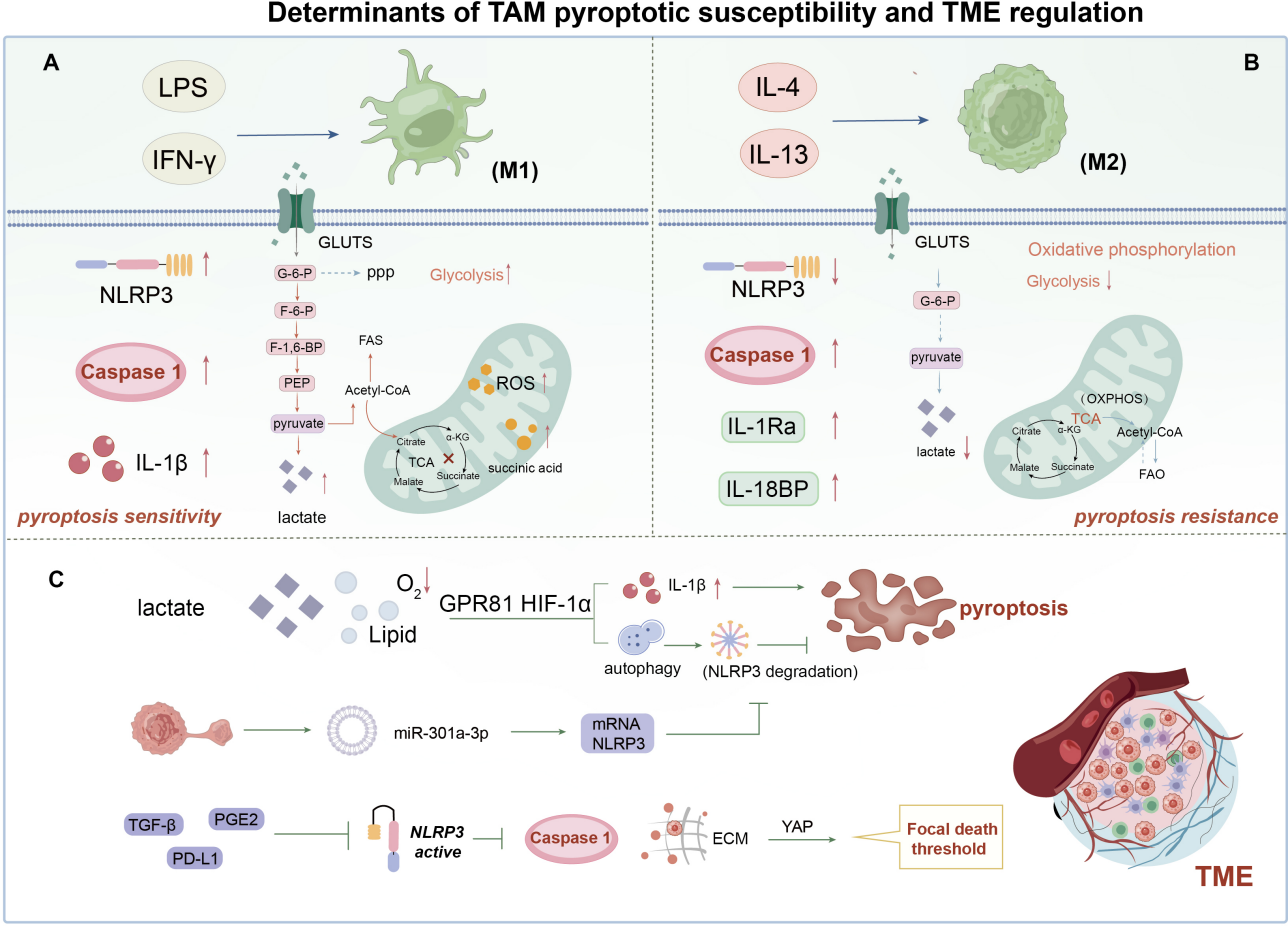
Determinants of TAM pyroptotic susceptibility and tumour-microenvironment regulation. **(A)** Pyroptosis-sensitive (M1-like) state. LPS plus IFN-γ stimulation upregulates NLRP3, caspase-1, and IL-1β. Metabolism is shifted toward aerobic glycolysis (G-6-P → F-6-P → F-1, 6-BP → PEP → pyruvate → lactate), with tricarboxylic acid (TCA) cycle disruption, succinate accumulation, and elevated mitochondrial ROS, which together lower the NLRP3 activation threshold. **(B)** Pyroptosis-resistant (M2-like) state. IL-4/IL-13 stimulation reduces NLRP3/caspase-1 expression while increasing IL-1Ra and IL-18BP. Metabolism shifts toward oxidative phosphorylation (OXPHOS) and fatty acid oxidation (FAO); glycolytic flux and lactate output are reduced. **(C)** TME imposes layered regulation on TAM pyroptosis. Three regulatory axes are shown. (i) *Metabolic*: lactate engages GPR81/HIF-1α to drive autophagic clearance of NLRP3; hypoxia and lipid overload further modulate IL-1β transcription. (ii) *Post-transcriptional*: exosomal miR-301a-3p represses NLRP3 mRNA, exemplifying ncRNA-mediated fine-tuning. (iii) *Soluble factors and biophysical cues*: TGF-β, PGE2, and PD-L1 inhibit NLRP3 activation, and ECM stiffness signalling through Yes-associated protein (YAP) raises the “focal death threshold” that gates TAM pyroptosis within the TME (right). Colour code: red arrows = stimulatory; green/blunt-ended lines = inhibitory; blue arrows = signal transduction. ECM, extracellular matrix; F-1, 6-BP, fructose-1, 6-bisphosphate; F-6-P, fructose-6-phosphate; FAO, fatty acid oxidation; G-6-P, glucose-6-phosphate; GLUT, glucose transporter; GPR81, G protein–coupled receptor 81; HIF-1α, hypoxia-inducible factor 1-alpha; IFN-γ, interferon gamma; IL-1Ra, IL-1 receptor antagonist; IL-18BP, IL-18 binding protein; M1, classically activated macrophage; M2, alternatively activated macrophage; ncRNA, non-coding RNA; NLRP3, NLR family pyrin domain–containing 3; OXPHOS, oxidative phosphorylation; PD-L1, programmed death-ligand 1; PEP, phosphoenolpyruvate; PGE2, prostaglandin E2; ROS, reactive oxygen species; TCA, tricarboxylic acid cycle; TGF-β, transforming growth factor beta; TME, tumour microenvironment; YAP, Yes-associated protein.

The M1/M2 dichotomy remains a useful heuristic but should not be treated as a mechanistic determinant of TAM pyroptosis. **Figures 2A, B** summarize the multidimensional view of TAM pyroptotic competence as the integrated output of TLR priming, IFN signaling, DAMP exposure, and phagocytic burden, with metabolism as a modulator rather than a driver.

#### 2.2.2 Regulation of macrophage pyroptosis by the tumor microenvironment

The TME imposes layered regulation on macrophage pyroptosis, with both tumor-promoting (immune evasion) and therapy-relevant consequences (12, 13): (i) metabolic reprogramming, in which tumor-derived lactate engages GPR81 and drives HIF-1α–dependent autophagic clearance of NLRP3, while hypoxia-stabilized HIF-1α exerts context-dependent effects on IL-1β transcription (12, 13) (see **Figure 2C**, top axis); (ii) post-transcriptional modulation by non-coding RNAs (ncRNAs) (see **Figure 2C**, middle axis: miR-301a-3p), including microRNAs (miRNAs), long non-coding RNAs (lncRNAs), and circular RNAs (circRNAs), which fine-tune NLRP3 inflammasome priming through targeting of NLRP3 itself or its upstream regulators (40); and (iii) soluble immunosuppressive factors (TGF-β, PD-L1, PGE2) together with biophysical cues from a stiffened, collagen-rich extracellular matrix, which together raise the pyroptotic activation threshold and contribute to tumor immune evasion (see **Figure 2C**, bottom axis: TGF-β/PGE2/PD-L1 → NLRP3).

Among these, metabolic regulation is particularly tightly coupled to the tumor-specific milieu. Glucose depletion, lactate accumulation, decreased oxygen tension, and lipid overload jointly modulate macrophage pyroptosis. Lactate-driven histone lactylation has been reported to promote a homeostatic gene-expression program in macrophages, representing an emerging metabolism-to-transcription axis (12). Beyond lactate–GPR81 signaling, the broader family of proton-sensing G protein–coupled receptors (GPCRs) (e.g., GPR4, GPR65, GPR68/OGR1) detects the acidic extracellular pH characteristic of solid tumors and may further modulate macrophage activation status. The hypoxic environment, through HIF-1α stabilization, may either upregulate IL-1β transcription or, depending on hypoxia severity and duration, redirect cellular metabolism in a manner that suppresses inflammasome output (13).

Immunosuppressive cytokines constitute an additional regulatory layer. IL-10, secreted by tumor cells and regulatory immune populations, exerts anti-inflammatory effects in part through metabolic reprogramming of macrophages, shifting cellular metabolism away from the glycolytic state that typically favors inflammasome activation (41).

### 2.3 Morphological and biochemical features of pyroptosis

Pyroptosis exhibits a morphological and biochemical signature that distinguishes it from apoptosis and necrosis. Morphologically, pyroptotic cells display cell swelling rather than the shrinkage typical of apoptosis, characteristic membrane blebs preceding plasma membrane rupture, and chromatin condensation without the apoptotic DNA ladder pattern; intracellular contents, including relatively intact organelles, are released into the extracellular space (2). Live-cell imaging further reveals substantial heterogeneity in the time course from GSDMD pore formation to complete lysis, reflecting differences in pore density and ESCRT-mediated repair efficiency (17, 18).

Biochemical readouts include: caspase-1/4/5/11 activation (fluorescent-labeled inhibitor of caspases (FLICA) assay or activity-specific antibodies); cleaved GSDMD-NT detected by Western blot, regarded as the most specific molecular marker (42); mature IL-1β and IL-18 in the supernatant by enzyme-linked immunosorbent assay (ELISA); lactate dehydrogenase (LDH) release as a sensitive but non-specific lytic marker; ASC speck formation by immunofluorescence; and a flow cytometry profile of propidium iodide (PI)–positive/Annexin V–low or negative, which discriminates pyroptosis from early apoptosis (Annexin V–positive/PI-negative) (42).

*In vivo* detection in tumor tissue is technically demanding because pyroptotic debris is rapidly phagocytosed by neighboring cells, rendering pyroptotic cells under-represented in fixed sections. Multiplex immunofluorescence (e.g., cluster of differentiation 68 (CD68) + GSDMD-NT + IL-1β co-staining) and parallel measurement of soluble pyroptosis-associated DAMPs (HMGB1, S100 proteins, IL-1β, IL-18, cell-free DNA (cfDNA)) in serum or tumor interstitial fluid are commonly used as complementary readouts that indirectly reflect *in vivo* pyroptotic activity (43).

## 3 Mechanisms of pyroptosis-induced cytokine storm

Terminology and scope. Before proceeding, the term “cytokine storm” is defined as used throughout this review. *Classical (systemic) cytokine storm* refers to pathological, system-wide immune hyperactivation with sustained elevation of multiple pro-inflammatory cytokines (IL-1β, IL-6, IL-18, TNF-α, interferon gamma (IFN-γ)) in the circulation, producing clinical syndromes such as CAR-T–associated CRS, sepsis, hemophagocytic lymphohistiocytosis/macrophage activation syndrome (HLH/MAS), and severe viral infection; hemodynamic instability, capillary leak, and multi-organ dysfunction are hallmarks (15, 44). *Localized micro-cytokine storm (LMCS)*, the term used in this review, refers to spatially confined (sub-millimeter to millimeter scale), transient, high-amplitude cytokine release within discrete TME inflammatory hotspots, without necessarily producing systemic symptoms. The concept is supported by spatial transcriptomic studies showing clustered, non-uniform distribution of pyroptotic TAMs and cytokine-expressing cells within human tumors (45).

Justification and caveat. The “storm” metaphor is retained because (i) the kinetic profile within pyroptotic hotspots, with rapid onset, high amplitude, self-amplification, and subsequent resolution or chronicity, appears to mirror the dynamic signature of systemic storms at reduced spatial scale, and (ii) the molecular drivers (NLRP3, GSDMD, IL-1β, IL-18, IL-6, TNF-α, HMGB1) overlap substantially with those in systemic CRS, permitting mechanistic read-across. We do not claim that LMCS is pathophysiologically equivalent to systemic CRS; whether molecular drivers, resolution kinetics, and therapeutic sensitivities translate directly between scales remains an open empirical question. Throughout Sections 3–6, “cytokine storm” without qualification refers to LMCS unless explicitly stated.

### 3.1 Cascade release of inflammatory mediators

Macrophage pyroptosis triggers a multi-layered cytokine cascade through temporally and spatially organized signaling. Rather than the passive consequence of cell death implied by older models, pyroptosis is now understood as an actively programmed inflammatory process: GSDMD pores enable selective pro-inflammatory cytokine secretion before complete membrane lysis, after which DAMPs released upon lysis amplify and broaden the response.

**Figure 3:**
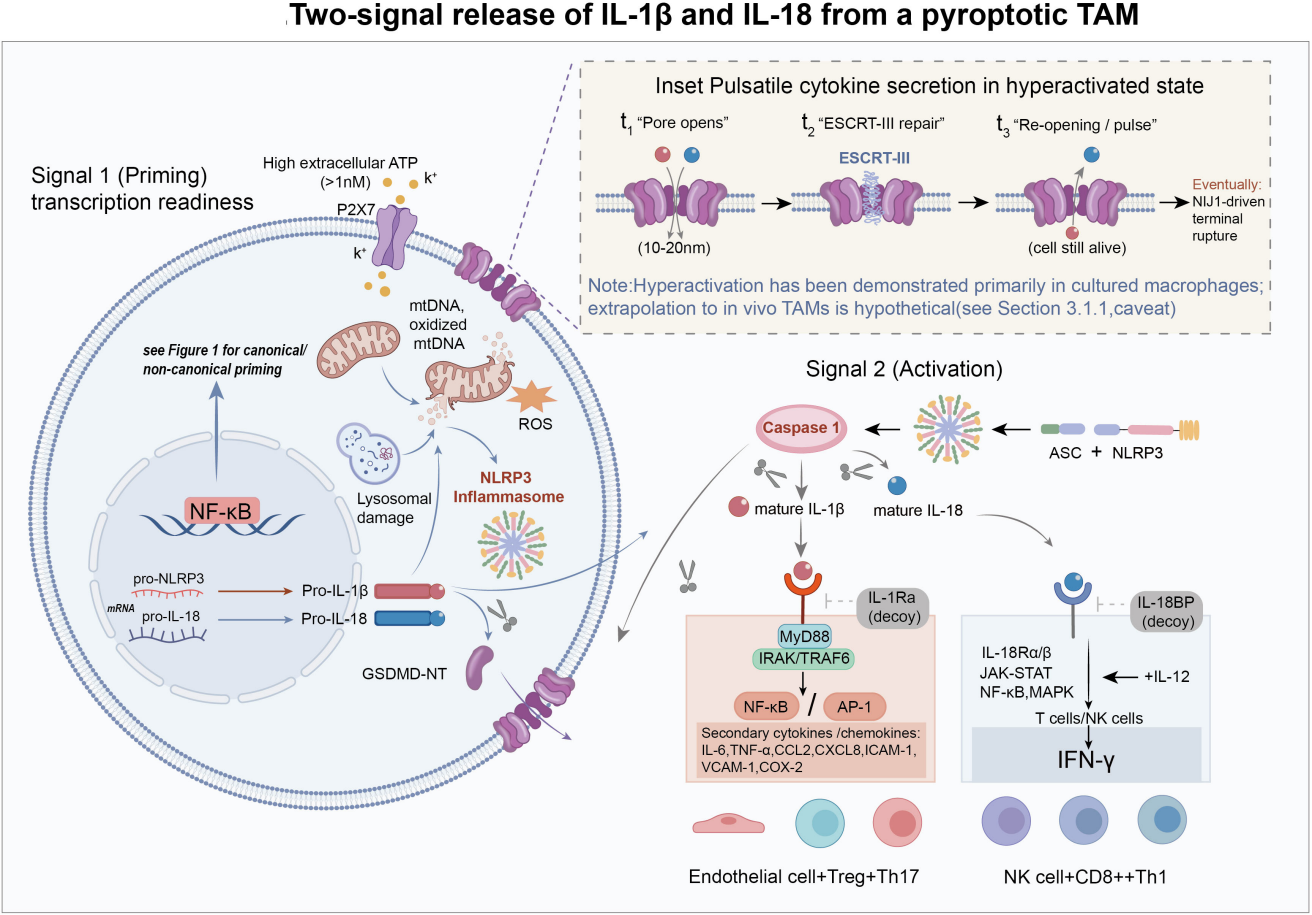
Two-signal release of IL-1β and IL-18 from pyroptotic TAMs. The schematic depicts the obligate two-signal mechanism by which the two core inflammasome-derived cytokines are generated and released by an individual TAM. Signal 1 (“priming”, left): PAMP/DAMP recognition by TLRs activates NF-κB, transcriptionally upregulating NLRP3, pro-IL-1β, and pro-IL-18. Signal 2 (“inflammasome trigger”, right): activation cues, including ATP/P2X7-induced K^+^ efflux, oxidised mtDNA, lysosomal damage, and ROS, drive NLRP3 inflammasome assembly via ASC, leading to caspase-1 activation and cleavage of pro-IL-1β/pro-IL-18 to their mature forms. Pore dynamics (top inset): GSDMD-NT pores permit selective cytokine release; ESCRT-III–mediated resealing of sub-lytic damage produces a pulsatile rather than continuous secretion profile in the “hyperactivation” state, prior to NINJ1-driven terminal rupture. Downstream signalling (bottom): released IL-1β engages IL-1R1/MyD88/IRAK/TRAF6, activating NF-κB/AP-1 to induce secondary cytokines and chemokines (IL-6, TNF-α, CCL2, CXCL8); released IL-18 engages IL-18Rα/β, activating JAK/STAT, NF-κB, and MAPK, and synergising with IL-12 to drive IFN-γ. IL-1Ra and IL-18BP are depicted as decoy receptors imposing negative feedback. The “hyperactivation”/pulsatile-secretion phenotype has been demonstrated primarily in cultured macrophages and is best regarded as a mechanistic hypothesis when extrapolated to human TAMs (see Section 3.1.1, caveat). AP-1, activator protein 1; ASC, apoptosis-associated speck-like protein containing a CARD; ATP, adenosine triphosphate; CCL2, C-C motif chemokine ligand 2; CXCL8, C-X-C motif chemokine ligand 8; DAMPs, damage-associated molecular patterns; ESCRT-III, endosomal sorting complexes required for transport III; GSDMD-NT, gasdermin D N-terminal fragment; IFN-γ, interferon gamma; IL, interleukin; IL-1R1, IL-1 receptor type 1; IL-1Ra, IL-1 receptor antagonist; IL-18BP, IL-18 binding protein; IRAK, IL-1 receptor–associated kinase; JAK, Janus kinase; MAPK, mitogen-activated protein kinase; mtDNA, mitochondrial DNA; MyD88, myeloid differentiation primary response 88; NF-κB, nuclear factor κB; NLRP3, NLR family pyrin domain–containing 3; P2X7, purinergic receptor P2X 7; PAMPs, pathogen-associated molecular patterns; STAT, signal transducer and activator of transcription; TAM, tumour-associated macrophage; TLR, Toll-like receptor; TNF-α, tumour necrosis factor alpha; TRAF6, TNF receptor–associated factor 6.

**Figure 4:**
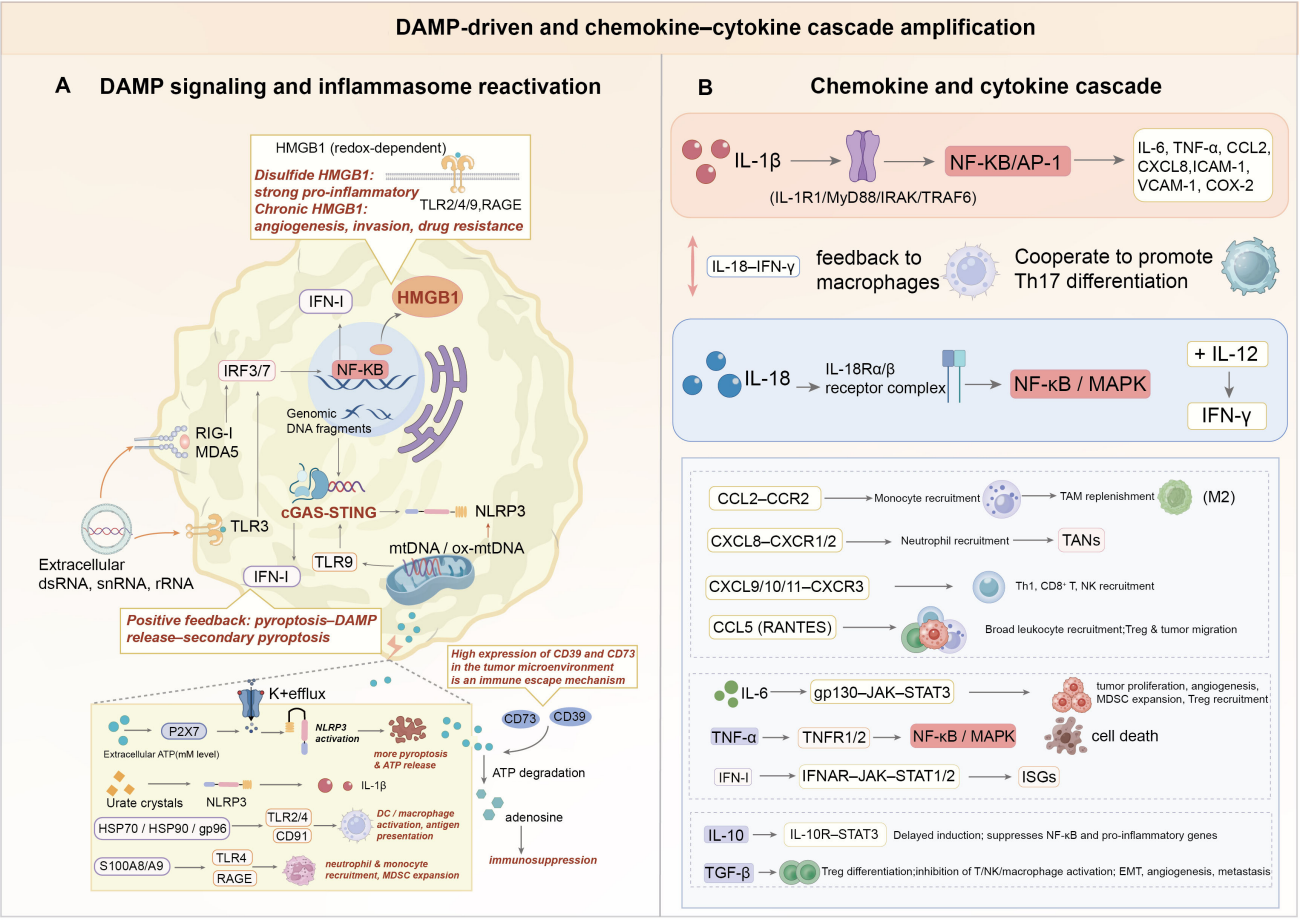
DAMP-driven inflammasome reactivation and chemokine–cytokine cascade amplification. **(A)** DAMP signalling and inflammasome reactivation. DAMPs released by pyroptotic cells engage multiple sensing systems on neighbouring TAMs. HMGB1 (redox-dependent: disulfide form pro-inflammatory; chronic exposure linked to angiogenesis, invasion, and drug resistance) signals via TLR2/4/9 and RAGE. Cytosolic genomic DNA fragments and mtDNA/oxidised mtDNA activate cGAS–STING and TLR9, while extracellular dsRNA/snRNA/rRNA are recognised by TLR3 and RIG-I/MDA5; the resulting IRF3/7 and NF-κB activation drives type I interferon (IFN-I) induction and feeds back to license further NLRP3 activation. The dashed yellow box outlines the *pyroptosis → DAMP release → secondary pyroptosis* positive-feedback loop: high extracellular ATP engages P2X7 to drive K^+^ efflux and NLRP3 activation, amplifying further pyroptosis and ATP release. CD39/CD73 (red box) hydrolyse ATP to immunosuppressive adenosine and represent a key tumour immune-escape mechanism. Urate crystals (NLRP3 → IL-1β), HSP70/HSP90/gp96 (TLR2/4/CD91 → DC and macrophage activation), and S100A8/A9 (TLR4, RAGE → neutrophil/monocyte recruitment, MDSC expansion) are additional DAMP inputs. **(B)** Chemokine–cytokine cascade. IL-1β engages IL-1R1/MyD88/IRAK/TRAF6 → NF-κB/AP-1, inducing IL-6, TNF-α, CCL2, CXCL8, ICAM-1, VCAM-1, and cyclooxygenase-2 (COX-2). IL-18 engages IL-18Rα/β → NF-κB/MAPK; in synergy with IL-12 it drives IFN-γ; bidirectional IL-18/IFN-γ feedback to macrophages and cooperative promotion of Th17 differentiation are indicated. Four chemokine axes selectively recruit distinct immune populations: CCL2–CCR2 (monocytes → M2-skewed TAM replenishment); CXCL8–CXCR1/2 (neutrophils → tumour-associated neutrophils, TANs); CXCL9/10/11–CXCR3 (Th1/CD8^+^ T/NK cells); CCL5 (broad leukocyte recruitment including Tregs and tumour-cell migration). Downstream effectors: IL-6 → gp130/JAK/STAT3 (tumour proliferation, angiogenesis, MDSC expansion, Treg recruitment); TNF-α → TNFR1/2 → NF-κB/MAPK (cell death); IFN-I → IFNAR/JAK/STAT1/2 → interferon-stimulated genes (ISGs). IL-10 → IL-10R/STAT3 (delayed induction; suppresses NF-κB and pro-inflammatory genes) and TGF-β (Treg differentiation, T/NK/macrophage inhibition, EMT, angiogenesis, metastasis) provide negative feedback. *Colour code:* red arrows = stimulatory; dashed boxes = positive-feedback loops; blunt-end (T-shape) = inhibition. AP-1, activator protein 1; CCL/CXCL, C-C/C-X-C motif chemokine ligand; CCR/CXCR, C-C/C-X-C motif chemokine receptor; cGAS–STING, cyclic GMP–AMP synthase/stimulator of interferon genes; COX-2, cyclooxygenase-2; DAMPs, damage-associated molecular patterns; DC, dendritic cell; dsRNA, double-stranded RNA; EMT, epithelial–mesenchymal transition; gp130, glycoprotein 130; HMGB1, high-mobility group box 1; HSP, heat shock protein; ICAM-1, intercellular adhesion molecule 1; IFNAR, type I interferon receptor; IFN-I, type I interferon; IFN-γ, interferon gamma; IRAK, IL-1 receptor–associated kinase; IRF, interferon regulatory factor; ISGs, interferon-stimulated genes; JAK, Janus kinase; MDA5, melanoma differentiation–associated protein 5; MDSC, myeloid-derived suppressor cell; mtDNA, mitochondrial DNA; MyD88, myeloid differentiation primary response 88; NF-κB, nuclear factor κB; NK, natural killer cell; ox-mtDNA, oxidised mitochondrial DNA; P2X7, purinergic receptor P2X 7; RAGE, receptor for advanced glycation end-products; RIG-I, retinoic acid–inducible gene I; rRNA, ribosomal RNA; S100A8/A9, S100 calcium-binding proteins A8/A9; snRNA, small nuclear RNA; STAT, signal transducer and activator of transcription; TANs, tumour-associated neutrophils; TGF-β, transforming growth factor beta; Th17, T helper 17; TLR, Toll-like receptor; TNF-α, tumour necrosis factor alpha; TNFR, TNF receptor; TRAF6, TNF receptor–associated factor 6; Tregs, regulatory T cells; VCAM-1, vascular cell adhesion molecule 1.

**Table 2** summarizes the principal mediators of the pyroptosis-driven localized cytokine storm — primary cytokines (IL-1β, IL-18), DAMPs (HMGB1, ATP, mtDNA, S100A8/A9), and secondary cytokines/chemokines (IL-6, TNF-α, CCL2, CXCL8, CXCL9/10/11) — with their principal source, key receptors, dominant downstream effects, and section cross-references. Throughout §3.1.1–3.1.3, individual mediators are discussed only at the level of mechanistic logic, with quantitative detail consolidated in **Table 2**. **Figure 3** zooms in on a single pyroptotic TAM to show how the two core cytokines, IL-1β and IL-18, are produced and released through a strictly two-signal mechanism. Signal 1 (left, “priming”): engagement of TLRs by PAMPs/DAMPs activates NF-κB, which transcriptionally upregulates NLRP3, pro-IL-1β, and pro-IL-18, establishing the inflammasome-readiness pool. Signal 2 (right, “inflammasome trigger”): ATP/K^+^ efflux/oxidized mtDNA/lysosomal damage trigger NLRP3 inflammasome assembly and caspase-1 activation, which cleaves the precursors into mature IL-1β and IL-18. Top inset depicts the GSDMD pore at the plasma membrane, alternating between transient ESCRT-III–mediated sub-lytic resealing and progressive enlargement; this dynamic underlies the proposed pulsatile rather than continuous IL-1β release in the “hyperactivation” state. Bottom arrows trace downstream signaling: IL-1β engages IL-1R1 → MyD88 → NF-κB/AP-1 to induce a secondary wave (IL-6, TNF-α, CCL2, CXCL8); IL-18 engages IL-18Rα/β → JAK/STAT, NF-κB, MAPK, and synergizes with IL-12 to drive IFN-γ. The figure thus serves as the mechanistic anchor for Section 3.1.1; subsequent amplification by DAMPs and chemokine networks is shown in **Figure 4**.

**Table 2:** Key mediators of pyroptosis-driven localized cytokine storm in the tumor microenvironment.

Mediator	Class	Principal source in TME	Key receptor(s)	Dominant downstream effect	Section
IL-1β	Pro-inflammatory cytokine	Pyroptotic TAM (dominant); pyroptotic tumor cells (secondary)	IL-1R1/IL-1RAcP	NF-κB and AP-1 activation; cascade amplification of IL-6, TNF-α, CCL2, CXCL8; dual acute pro-immune vs chronic pro-tumor roles	3.1.1
IL-18	Pro-inflammatory cytokine	Pyroptotic TAM	IL-18Rα/IL-18Rβ	IFN-γ induction (with IL-12); NK and CD8^+^ T cell cytotoxicity; T cell exhaustion under chronic exposure	3.1.1
HMGB1	Nuclear DAMP	Pyroptotic tumor cell (primary, ICD); pyroptotic TAM (secondary)	TLR2/TLR4, RAGE	Acute: DC maturation and antigen cross-presentation; chronic: angiogenesis, EMT, metastasis (redox-state dependent)	3.1.2
ATP	Metabolic DAMP	Any pyroptotic cell	P2X7, P2Y2	NLRP3 priming via K^+^ efflux; DC recruitment/activation; self-amplifying pyroptosis loop	3.1.2
mtDNA/ox-mtDNA	Nucleic-acid DAMP	Pyroptotic cell (mitochondrial origin)	TLR9, cGAS–STING, NLRP3 (direct binding)	Type I IFN induction; NLRP3 reinforcement; pyroptosis-to-pyroptosis feed-forward loop	3.1.2
S100A8/A9 (calprotectin)	Alarmin DAMP	Neutrophil, TAM	TLR4, RAGE	Myeloid recruitment; pre-metastatic niche formation; ECM remodeling	3.1.2
IL-6	Secondary cytokine	TAM, tumor cell, stromal cell	IL-6R/gp130 (classical and trans-signaling)	STAT3 activation; tumor proliferation, angiogenesis, immunosuppression; CRS amplification	3.1.3
TNF-α	Secondary cytokine	TAM, activated T cell	TNFR1, TNFR2	NF-κB/MAPK activation; vascular permeability; context-dependent survival vs apoptosis	3.1.3
CCL2 (MCP-1)	Chemokine (CC)	TAM, tumor cell	CCR2	Monocyte recruitment; TAM pool replenishment; M2-like skewing via JAK2–STAT3	3.1.3
CXCL8 (IL-8)	Chemokine (CXC)	TAM, tumor cell, endothelium	CXCR1, CXCR2	Neutrophil recruitment in acute inflammation; endothelial migration; pro-angiogenic	3.1.3
CXCL9/10/11	Chemokine (CXC, IFN-induced)	IFN-γ–stimulated TAM, endothelium	CXCR3	Th1, CD8^+^ T, and NK cell recruitment; “hot tumor” phenotype; improved anti-PD-1/PD-L1 response	3.1.3

AP-1, activator protein 1; CCL, CC chemokine ligand; CCR, CC chemokine receptor; cGAS, cyclic GMP–AMP synthase; CRS, cytokine release syndrome; CXCL, CXC chemokine ligand; CXCR, CXC chemokine receptor; DAMP, damage-associated molecular pattern; DC, dendritic cell; ECM, extracellular matrix; EMT, epithelial–mesenchymal transition; gp130, glycoprotein 130; ICD, immunogenic cell death; IFN, interferon; IL, interleukin; IL-1RAcP, IL-1 receptor accessory protein; IL-1R1, IL-1 receptor type 1; JAK, Janus kinase; MAPK, mitogen-activated protein kinase; MCP-1, monocyte chemoattractant protein 1; mtDNA, mitochondrial DNA; NF-κB, nuclear factor kappa B; NK, natural killer; NLRP3, NLR family pyrin domain containing 3; ox-mtDNA, oxidized mtDNA; PD-1/PD-L1, programmed death-1/ligand-1; P2X7/P2Y2, purinergic receptors; RAGE, receptor for advanced glycation end-products; STAT, signal transducer and activator of transcription; STING, stimulator of interferon genes; TAM, tumor-associated macrophage; TLR, toll-like receptor; TME, tumor microenvironment; TNF, tumor necrosis factor; TNFR, TNF receptor.

#### 3.1.1 IL-1β and IL-18: core inflammatory mediators

##### 3.1.1.1 IL-1β: two-signal regulation and dynamic secretion

IL-1β is among the most important pro-inflammatory cytokines released during pyroptosis. Its production requires two sequential signals (**Figure 3**, left arm): Signal 1 (TLR–PAMP recognition driving NF-κB–dependent transcription of pro-IL-1β and pro-IL-18) (36), and Signal 2 (NLRP3 inflammasome assembly and caspase-1–mediated cleavage of the precursors). GSDMD pores then mediate cytokine release.

*In vitro*, a state termed “hyperactivation” has been described in which mature IL-1β is actively secreted through GSDMD pores in viable, partially intact macrophages prior to overt lysis (22) (see top inset of **Figure 3**). ESCRT-III–mediated membrane repair can transiently reseal these pores, producing pulsatile rather than continuous IL-1β release (18). *Caveat:* hyperactivation has been demonstrated almost exclusively in cultured macrophages. Direct evidence that pyroptotic TAMs in human tumors transit through a prolonged hyperactive state, rather than rapidly proceeding to NINJ1-mediated lytic rupture (17), is currently lacking. Throughout this review, descriptions of “pulsatile” or “prolonged” IL-1β secretion should therefore be interpreted as a mechanistic hypothesis extrapolated from *in vitro* systems, not as an established *in vivo* property of TAMs.

IL-1β released into the TME engages IL-1R1, activating NF-κB and activator protein 1 (AP-1) to induce a downstream wave of cytokines (IL-6, TNF-α) and chemokines (CCL2, CXCL8) (46, 47). Functionally, IL-1β has well-documented dual roles: short-term, high-amplitude exposure may augment NK and CD8^+^ T cell cytotoxicity and dendritic cell antigen presentation, whereas chronic low-grade IL-1β signaling has been linked to angiogenesis, epithelial–mesenchymal transition, and chemoresistance (48). The CANTOS trial provided the most influential clinical evidence for the pro-tumorigenic role of chronic IL-1β: in cardiovascular disease patients, IL-1β blockade with canakinumab was associated with substantially reduced lung cancer incidence and mortality (49, 50), a finding that has catalyzed clinical exploration of IL-1β inhibition in oncology.

##### 3.1.1.2 IL-18: an immediate-response cytokine

Unlike IL-1β, pro-IL-18 is constitutively expressed in many cell types, allowing rapid release upon inflammasome activation without requiring transcriptional priming. Mature IL-18 signals through the IL-18Rα/β complex to activate Janus kinase–signal transducer and activator of transcription (JAK–STAT), NF-κB, and mitogen-activated protein kinase (MAPK) pathways (51). Its most prominent function is potent induction of IFN-γ, particularly in synergy with IL-12 (52) (see right downstream branch of **Figure 3**).

In acute settings, IL-18 enhances NK and CD8^+^ T cell cytotoxicity (53), whereas sustained IL-18 exposure has been linked to T cell exhaustion and upregulation of inhibitory checkpoints (PD-1, T-cell immunoglobulin and mucin domain–containing 3 (TIM-3), lymphocyte-activation gene 3 (LAG-3)) (54). IL-18 activity is restrained by IL-18 binding protein (IL-18BP), a high-affinity soluble decoy receptor (51). IL-18BP is reported to be overexpressed in several tumor types and may represent an immune evasion mechanism (55), providing a rationale for IL-18BP–resistant IL-18 analogs.

##### 3.1.1.3 Synergy

IL-1β and IL-18 reinforce one another through several positive feedback mechanisms: IL-1β upregulates IL-18 receptor expression; IL-18-driven IFN-γ supports M1-like macrophage polarization, and these macrophages may themselves have a stronger pyroptosis tendency, completing the loop; both cytokines together promote T helper 17 (Th17) differentiation (48). Preclinical work suggests that combined IL-1β plus IL-18 blockade may inhibit tumor progression more effectively than blocking either alone (56), pointing toward dual-target strategies.

#### 3.1.2 Damage-associated molecular patterns

Loss of plasma membrane integrity during pyroptosis releases intracellular molecules that act as endogenous danger signals (**Figure 4**). Key DAMPs include HMGB1, nucleic acids, ATP, urate, heat shock proteins, and S100 proteins, which converge on PRRs and the inflammasome to amplify the local response.

Cell-type scope: bidirectional — primary high-amplitude release from pyroptotic tumor cells (ICD); secondary cascade-amplifying release from pyroptotic TAMs.

##### 3.1.2.1 HMGB1

HMGB1 is among the most thoroughly studied DAMPs and is released both from pyroptotic tumor cells (primary, large quantities accompanying immunogenic cell death) and from pyroptotic TAMs (secondary, cascade-amplifying) (57). Its biological activity is redox-state dependent: the disulfide form binds TLR4–MD2 with high affinity and is the dominant pro-inflammatory species, the fully reduced form is chemotactic, and the fully oxidized form is inactive (58). Acutely released HMGB1 has been linked to dendritic cell maturation and cross-presentation through TLR4, a pathway with recognized relevance to anthracycline chemotherapy efficacy (59). Chronic HMGB1 exposure may instead promote angiogenesis, EMT, and metastasis through receptor for advanced glycation end-products (RAGE) (57) (see **Figures 4A, top**-left).

##### 3.1.2.2 Nucleic acid DAMPs

Cytoplasmic genomic DNA released during pyroptosis activates the cGAS–STING pathway and induces type I interferon (IFN-I) (60). Mitochondrial DNA (mtDNA) is particularly immunogenic because of its bacterial-like unmethylated cytosine–phosphate–guanine (CpG) motifs (TLR9) and dual cGAS–STING activation; oxidized mtDNA can directly bind NLRP3, establishing a pyroptosis → DAMP → secondary pyroptosis loop (21). Extracellular RNA species are sensed by TLR3, retinoic acid–inducible gene I (RIG-I), and melanoma differentiation–associated protein 5 (MDA5) to drive interferon regulatory factor 3/7 (IRF3/7) and NF-κB activation (61) (see **Figures 4A**, middle).

##### 3.1.2.3 ATP and urate

Lysis of pyroptotic cells releases intracellular ATP into the extracellular space, where high concentrations engage purinergic receptor P2X7 (P2X7) receptors and trigger K^+^ efflux, a major NLRP3-activating signal, producing a self-amplifying pyroptosis–ATP–pyroptosis loop (62). This cascade is constrained by ectonucleoside triphosphate diphosphohydrolase 1 (CD39) and ecto-5′-nucleotidase (CD73), which sequentially convert ATP to immunosuppressive adenosine; high CD39/CD73 expression has been described as an immune evasion mechanism in many tumors and is the basis for ongoing clinical inhibitor programs (63). Massive cell death also releases urate, which can crystallize and activate NLRP3, relevant in tumor lysis syndrome (64) (see **Figure 4A**, dashed box at bottom).

##### 3.1.2.4 HSPs and S100 proteins

HSPs (HSP70, HSP90, glycoprotein 96 (gp96)) released during pyroptosis can activate dendritic cells and macrophages via TLR2/TLR4 and cluster of differentiation 91 (CD91) (65); HSP–peptide complexes carry tumor antigens and have been explored as autologous tumor vaccines. The S100A8/A9 heterodimer (calprotectin) signals through TLR4 and RAGE and is a potent neutrophil and monocyte chemoattractant (66) (see **Figures 4A**, lower-left subbox).

#### 3.1.3 Chemokine and cytokine amplification

Primary signals (IL-1β, IL-18, DAMPs) trigger expression of secondary cytokines and chemokines that organize selective immune cell recruitment (see **Figure 4B**, axes 1–4) (67). Major axes include: CC chemokine ligand 2–CC chemokine receptor 2 (CCL2–CCR2), recruiting circulating monocytes that replenish the TAM pool and may be skewed toward M2-like phenotypes via Janus kinase 2–signal transducer and activator of transcription 3 (JAK2–STAT3), with documented importance in breast cancer metastasis (68); CXCL8 (IL-8)–CXCR1/2, driving rapid neutrophil recruitment in acute inflammation (69), with a role that appears more prominent in human disease than in mouse models, which lack a true CXCL8 ortholog; CXCL9/10/11–CXCR3, induced predominantly by IFN-γ and recruiting Th1, CD8^+^ T, and NK cells, with high activity correlating with “hot” tumor phenotypes and improved response to PD-1/PD-L1 blockade (70); and CCL5–CCR5, broadly recruiting T cells, monocytes, and eosinophils (71).

Among secondary cytokines (see **Figure 4B**, lower section), IL-6 is induced by IL-1β through NF-κB and signals via classical (membrane IL-6R) and trans-signaling (soluble IL-6R + glycoprotein 130 (gp130)) modes; the latter has been associated particularly with chronic inflammation and tissue damage (72, 73). IL-6/STAT3 signaling supports tumor proliferation, angiogenesis, and an immunosuppressive TME, and IL-6 receptor blockade with tocilizumab is U.S. Food and Drug Administration (FDA)–approved for severe CAR-T–induced CRS (74). TNF-α acts through TNF receptor 1/2 (TNFR1/2) to drive NF-κB, MAPK, and caspase signaling, with context-dependent roles in inflammation amplification, survival, or apoptosis (75). IFN-I, induced by nucleic acid DAMPs, supports antigen presentation and antiviral states (76), while IFN-γ from NK and T cells (under IL-12/IL-18 synergy) drives M1-like macrophage polarization and is central to anti-tumor immunity (77). Counter-regulatory cytokines IL-10 and TGF-β provide negative feedback (discussed in §3.3.2) (78, 79).

### 3.2 Amplification mechanisms

#### 3.2.1 Secondary pyroptosis and spatial propagation

**Figure 5:**
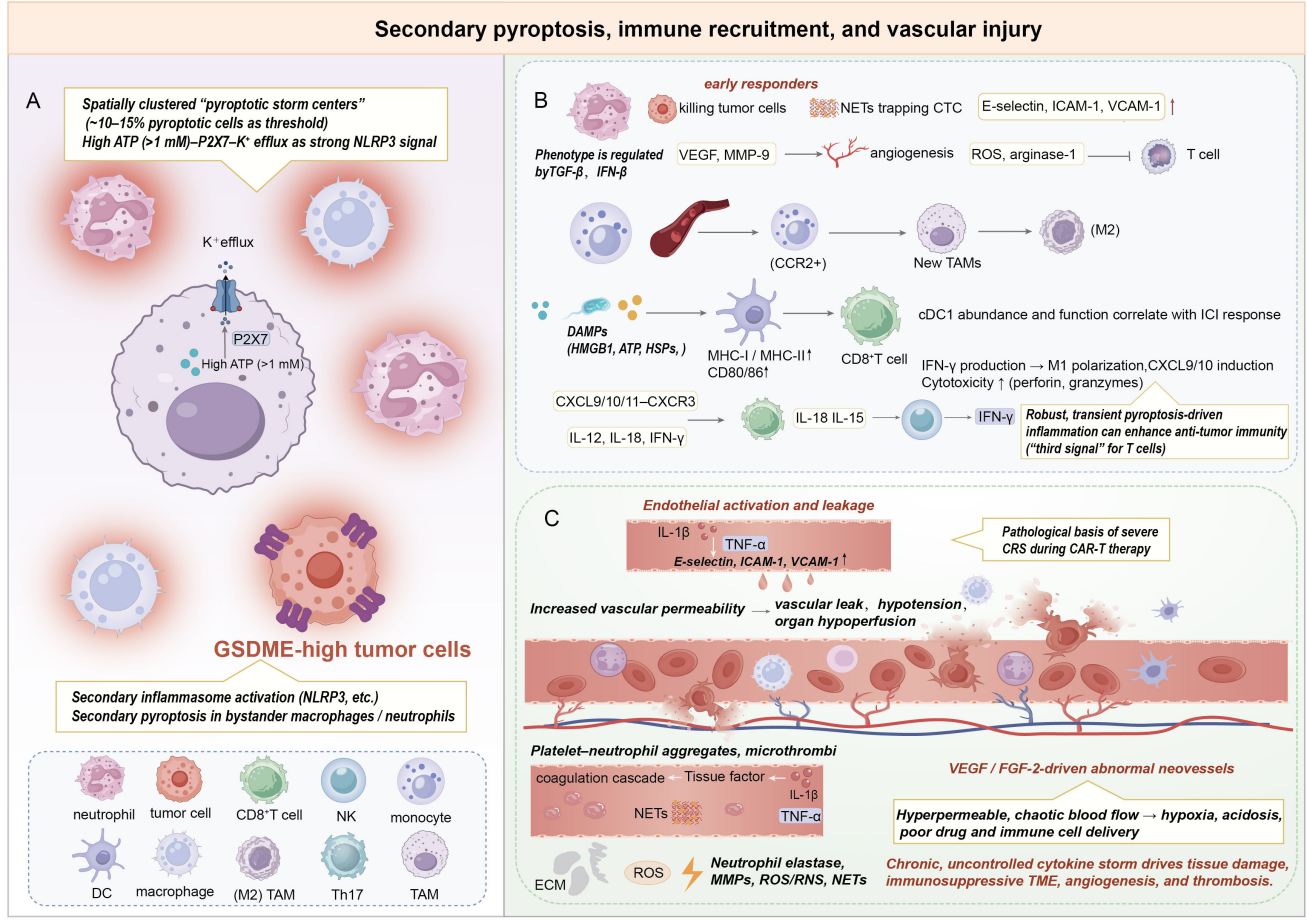
Secondary pyroptosis, immune-cell recruitment, and vascular injury within tumour inflammatory hotspots. **(A)** Spatial propagation of pyroptosis. Pyroptotic TAMs cluster spatially to form “pyroptotic storm centres, “ regions in which approximately 10–15% of cells are pyroptotic, a tentative threshold derived from murine 4T1 bioorthogonal experiments. High extracellular ATP (>1 mM) engages P2X7 receptors on neighbouring cells to drive K^+^ efflux, providing a strong NLRP3-activating signal that ignites secondary pyroptosis in bystander macrophages and neutrophils. GSDME-high tumour cells contribute a chemotherapy-relevant amplification route via caspase-3 cleavage. The cell-type legend at the lower left defines icons used in panels B and C (neutrophil, tumour cell, CD8^+^ T cell, NK, monocyte, DC, macrophage, M2 TAM, Th17, TAM). **(B)** Dynamic immune-cell recruitment. Sequential, chemokine-gradient-driven arrival of immune populations is depicted. Early-responding neutrophils (top) directly kill tumour cells, trap circulating tumour cells via NETs, and upregulate endothelial adhesion molecules (E-selectin, ICAM-1, VCAM-1); their phenotype is shaped by TGF-β and IFN-β, with TGF-β-conditioned “N2-like” neutrophils contributing to angiogenesis (VEGF, MMP-9) and T-cell suppression (ROS, arginase-1). CCR2^+^ monocytes are recruited and re-educated to M2-like TAMs. DAMPs (HMGB1, ATP, HSPs) drive cDC1 maturation, MHC-I/II and CD80/86 upregulation, and tumour-antigen cross-presentation to CD8^+^ T cells; cDC1 abundance and function correlate with response to immune checkpoint inhibitors. The IFN-γ axis (M1 polarisation, CXCL9/10 induction, increased perforin/granzyme cytotoxicity) provides the “third signal” for T-cell effector differentiation; IL-12/IL-18/IFN-γ jointly activate NK cells (IFN-γ output via IL-15/IL-18). **(C)** Vascular injury and tissue remodeling. (top) IL-1β/TNF-α activate endothelium, upregulating E-selectin, ICAM-1, and VCAM-1 and increasing permeability, yielding vascular leak, hypotension, and organ hypoperfusion; this constitutes the pathological basis of severe CAR-T–associated CRS. (middle) Platelet–neutrophil aggregates, microthrombi, NETs, neutrophil elastase, MMPs, and ROS/RNS damage matrix; tissue factor links inflammation to coagulation. *(bottom)* VEGF/FGF-2–driven abnormal neovessels are hyperpermeable and disorganised, producing hypoxia, acidosis, and impaired drug and immune-cell delivery. The bottom red text emphasises that, in the chronic/uncontrolled mode, this network drives tissue damage, immunosuppressive TME, angiogenesis, and thrombosis. ATP, adenosine triphosphate; CAR-T, chimeric antigen receptor T cell; CCR2, C-C motif chemokine receptor 2; cDC1, type 1 conventional dendritic cell; CRS, cytokine release syndrome; CTC, circulating tumour cell; DAMPs, damage-associated molecular patterns; DC, dendritic cell; ECM, extracellular matrix; FGF-2, fibroblast growth factor 2; GSDME, gasdermin E; HMGB1, high-mobility group box 1; HSPs, heat shock proteins; ICAM-1, intercellular adhesion molecule 1; IFN, interferon; IL, interleukin; MHC, major histocompatibility complex; MMP, matrix metalloproteinase; NETs, neutrophil extracellular traps; NK, natural killer cell; NLRP3, NLR family pyrin domain–containing 3; P2X7, purinergic receptor P2X 7; RNS, reactive nitrogen species; ROS, reactive oxygen species; TAM, tumour-associated macrophage; TGF-β, transforming growth factor beta; TNF-α, tumour necrosis factor alpha; VCAM-1, vascular cell adhesion molecule 1; VEGF, vascular endothelial growth factor.

Pyroptotic mediators activate inflammasomes in neighboring cells, producing cascade amplification. Extracellular ATP via P2X7-driven K^+^ efflux is a major secondary trigger (80); ox-mtDNA and urate crystals provide additional NLRP3-activating signals. Live imaging studies suggest a propagation radius of approximately 50–100 μm around individual pyroptotic cells. Spatial transcriptomic studies in human squamous cell carcinoma show pyroptotic macrophages clustered rather than uniformly distributed, forming local “cytokine storm centers” (see **Figure 5A**) (45). This pattern may reflect TME heterogeneity (hypoxia, low pH, locally elevated DAMPs).

Cell-type sensitivity to pyroptosis varies substantially. Professional immune cells (monocyte-derived macrophages, neutrophils) are highly responsive, whereas many epithelial-derived tumor cells express low GSDMD and appear relatively resistant (81). The observation that GSDME, cleaved by caspase-3, can convert apoptosis to pyroptosis in GSDME-high tumor cells reframed this picture and may help explain why some chemotherapeutics (cisplatin, doxorubicin) elicit strong inflammatory responses and potentiate immunotherapy (4). GSDME has been proposed as a candidate biomarker for chemotherapy–immunotherapy combinations.

A bioorthogonal approach in the 4T1 murine model reported that pyroptosis in approximately 10–15% of tumor cells was sufficient to trigger bystander activation and self-amplifying cascades (see threshold annotation in **Figure 5A**) (43). *Caveat:* this threshold derives from a single murine tumor model with accompanying in silico simulation; whether an analogous threshold operates in human tumors, and how it varies with tumor type, TAM density, and inflammasome competence, remains to be determined. Conceptually, the data suggest that therapeutic pyroptosis induction should aim to reach but not far exceed this threshold to maximize immune activation while limiting tissue damage (**Figure 2C**).

#### 3.2.2 Dynamic recruitment of immune cells

Chemokine gradients established within hours drive sequential immune cell recruitment (**Figure 5B**) (82). **Neutrophils** arrive first (within 2–4 h), recruited by CXCL8 and endothelial selectin upregulation (83). Newly recruited “N1-like” neutrophils may directly kill tumor cells and trap circulating tumor cells via neutrophil extracellular traps (NETs) (84), whereas TGF-β–conditioned “N2-like” tumor-associated neutrophils may instead support angiogenesis (vascular endothelial growth factor (VEGF)) and invasion (matrix metalloproteinase 9 (MMP-9)) (85); this plasticity appears shaped by the local cytokine balance.

Monocytes recruited via CCL2–CCR2 differentiate into macrophages that initially adopt M1-like, anti-tumor phenotypes (inducible nitric oxide synthase (iNOS), TNF-α, IL-12) but are progressively re-educated toward M2-like, immunosuppressive states (arginase 1 (Arg1), CD206, TGF-β) under TME signals such as colony-stimulating factor 1 (CSF-1), IL-4, and IL-10 (86). Blocking this transition is a central focus of TAM-targeted therapy. Dendritic cells mature in response to DAMPs and pro-inflammatory cytokines, with type 1 conventional dendritic cells (cDC1s; Batf3^+^ CD103^+^) playing a central role in cross-presenting tumor antigens and initiating CTL responses (87, 88). IFN-I in pyroptotic microenvironments enhances cDC1 antigen presentation and C-C chemokine receptor 7 (CCR7)–driven migration to draining lymph nodes. Subsequently, NK cells are activated by IL-15 and IL-18 (89), while CD8^+^ T cells receive the “third signal” required for full effector differentiation from IL-12, IL-18, and IFN-I (90).

#### 3.2.3 Vascular injury and tissue remodeling

IL-1β, TNF-α, and VEGF disrupt endothelial tight junctions, increasing vascular permeability (**Figures 5C**, top–middle–bottom panels respectively). In severe cytokine storms this manifests as hypotension, tissue hypoperfusion, and multi-organ dysfunction, a defining feature of severe CAR-T–associated CRS (44, 91). In chronic settings, persistent VEGF and fibroblast growth factor 2 (FGF-2) drive structurally abnormal, leaky tumor neovasculature that simultaneously supports tumor growth and impairs drug delivery (92). Bystander damage from ROS, nitric oxide (NO), neutrophil elastase, matrix metalloproteinases (MMPs), and NET-derived histones/DNA degrades the matrix and damages adjacent cells (92, 93); the resulting DAMPs feed back into the inflammatory cascade, a pattern termed “nonresolving inflammation” (93). IL-1β and TNF-α also induce tissue factor expression, linking inflammation to coagulation and contributing to disseminated intravascular coagulation (DIC) in severe CRS (94).

### 3.3 Self-limiting mechanisms

Despite the strong amplification potential of pyroptotic cascades, healthy organisms generally contain and resolve inflammation through layered negative feedback. Understanding these mechanisms provides targets for pro-resolution strategies.

#### 3.3.1 Natural cytokine inhibitors

**IL-1Ra** is an endogenous antagonist of IL-1R1 with affinity comparable to IL-1β but no agonist activity; effective blockade typically requires substantial molar excess, which may explain the high doses used clinically (36). IL-18BP binds free IL-18 with high affinity and prevents receptor engagement (51) Soluble cytokine receptors (soluble TNFR (sTNFR), soluble IL-6R (sIL-6R)) can either neutralize cytokines or extend their reach via trans-signaling, depending on context (73).

#### 3.3.2 Anti-inflammatory cytokines and glucocorticoids

Anti-inflammatory cytokine production is generally delayed relative to pro-inflammatory factors, ensuring acute responses initiate before resolution begins. IL-10 (produced by M2-like macrophages, Tregs, and exhausted T cells) signals through STAT3, induces suppressor of cytokine signaling 3 (SOCS3), and downregulates MHC II and co-stimulatory molecules (78). TGF-β suppresses T and NK cell effector functions, promotes Foxp3^+^ Treg differentiation, and is among the most potent endogenous immunosuppressors (79). Systemically, glucocorticoids induced via the hypothalamic–pituitary–adrenal (HPA) axis activate anti-inflammatory transcriptional programs (e.g., mitogen-activated protein kinase phosphatase 1 (MKP-1)) while repressing pro-inflammatory genes (95).

#### 3.3.3 Intracellular brakes and immune checkpoints

Intracellular regulators include the suppressor of cytokine signaling (SOCS) family, which terminates JAK–STAT signaling, and A20 (TNF-α–induced protein 3, TNFAIP3), a ubiquitin-editing enzyme that restrains NF-κB and inflammasome activity (96). Inhibitory checkpoint receptors (PD-1, cytotoxic T-lymphocyte–associated protein 4 (CTLA-4), TIM-3, LAG-3), upregulated after activation, maintain tolerance under physiological conditions but can be co-opted as immune evasion mechanisms in tumors, the basis of checkpoint blockade therapy (97).

#### 3.3.4 Efferocytosis and pro-resolving mediators

Phagocytic clearance of apoptotic cells and pyroptotic debris (efferocytosis) is an active anti-inflammatory process (37). Phosphatidylserine on dying cells engages TAM-family receptors (Tyro3, Axl, Mer; TAM receptors); phagocytes that have ingested apoptotic cells downregulate TNF-α and IL-1β and upregulate TGF-β, PGE2, and IL-10 (38) Resolution is also supported by a “lipid mediator class switch” from pro-inflammatory eicosanoids (prostaglandins, leukotrienes) to specialized pro-resolving mediators (SPMs) (lipoxins, resolvins, protectins, maresins) derived from ω-3 polyunsaturated fatty acids (98, 99). SPMs promote neutrophil apoptosis, enhance efferocytosis, and dampen pro-inflammatory cytokine production while preserving antimicrobial defense.

Two further concepts shape this regulatory landscape. Metabolic reprogramming distinguishes glycolytic M1-like macrophages from oxidative M2-like macrophages and is tightly coupled with anti-inflammatory function (100). Trained immunity describes innate-immune “memory” via epigenetic reprogramming (e.g., H3K4me3, H3K27ac), in which prior exposures (Bacillus Calmette–Guérin (BCG), β-glucan) augment subsequent inflammatory responsiveness (101). Circadian regulation of immunity adds another layer of temporal organization (102).

## 4 Dual role of cytokine storm in tumor progression

Macrophage pyroptosis–triggered cytokine storms appear to exhibit “double-edged sword” effects on tumor progression. Whether the net effect is pro- or anti-tumor seems to depend on inflammation intensity, duration, spatial distribution, and contextual TME conditions. This duality may help explain heterogeneous clinical responses and suggests that strategies that simply “promote” or “inhibit” inflammation are unlikely to be optimal, pointing instead toward individualized, precisely tuned modulation.

### 4.1 Pro-tumor effects

**Figure 6:**
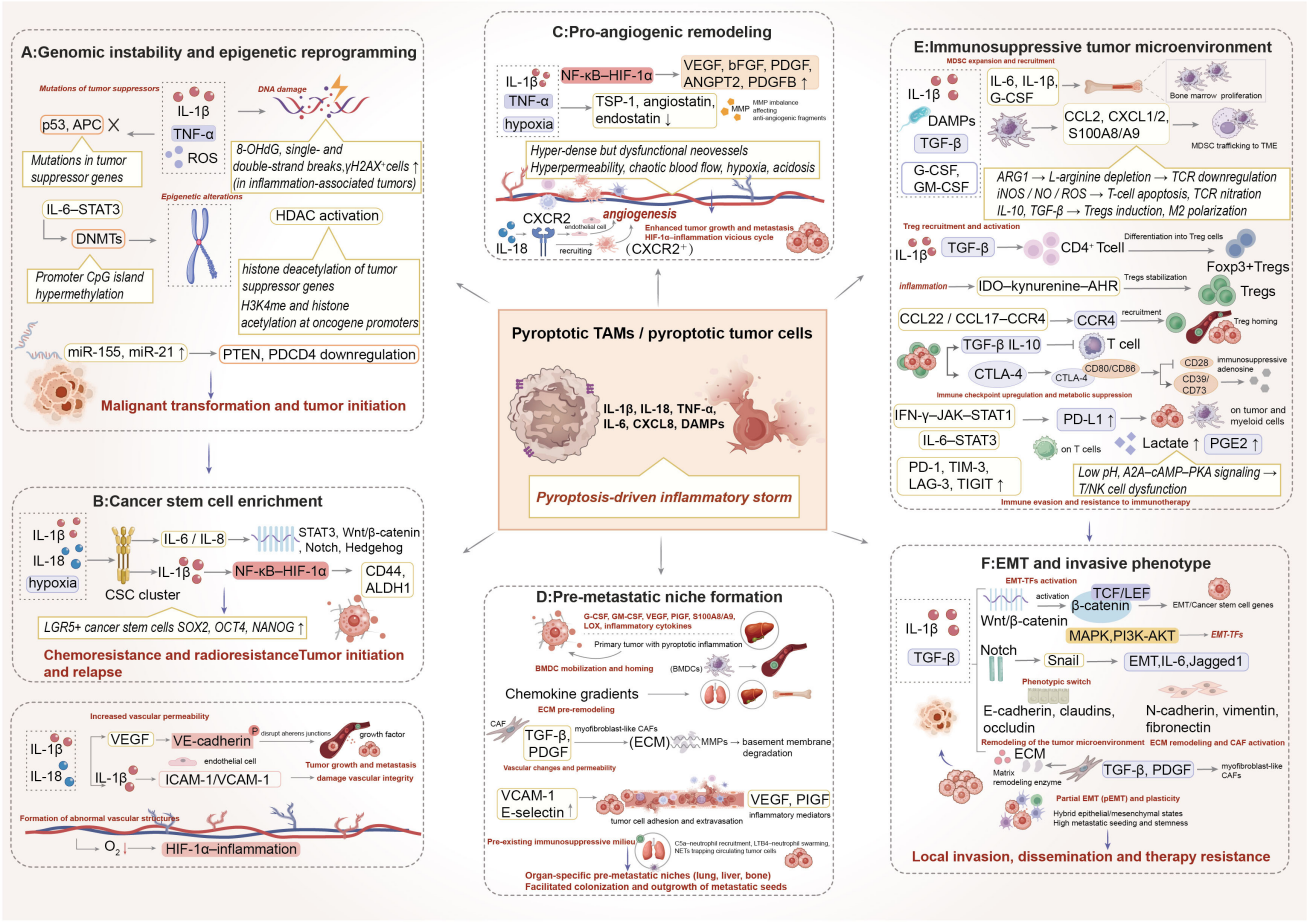
Chronic pyroptotic inflammation drives six interconnected pro-tumor mechanisms. Central node: pyroptotic TAMs and pyroptotic tumour cells release IL-1β, IL-18, TNF-α, IL-6, CXCL8, and DAMPs that, when chronically sustained at low grade, generate a “pyroptosis-driven inflammatory storm” radiating outward to six pro-tumour consequences. **(A)** Genomic instability and epigenetic reprogramming. IL-1β, TNF-α, and ROS produce DNA damage (8-OHdG, single- and double-strand breaks, γH2AX^+^ cells) and silence DNA repair, contributing to mutations in tumour suppressors (p53, adenomatous polyposis coli, APC). IL-6–STAT3 activates DNMTs, driving CpG-island hypermethylation of tumour-suppressor promoters. Inflammation-associated HDAC activity reshapes the histone landscape (deacetylation of tumour suppressors; H3K4 methylation/acetylation of oncogene promoters). Inflammation-induced miR-155 and miR-21 downregulate PTEN and PDCD4. The net effect is malignant transformation and tumour initiation. **(B)** Cancer stem cell enrichment. IL-1β, IL-18, and hypoxia synergise through IL-6/IL-8 → STAT3, Wnt/β-catenin, Notch, and Hedgehog signalling to upregulate CD44, ALDH1, LGR5 and pluripotency factors (SOX2, OCT4, NANOG), supporting chemoresistance, radioresistance, and tumour relapse. The lower sub-block depicts inflammation-driven vascular permeability changes (VEGF–VE-cadherin disruption; ICAM-1/VCAM-1 upregulation) and abnormal vasculature formation under HIF-1α–inflammation coupling. **(C)** Pro-angiogenic remodeling. IL-1β, TNF-α, and hypoxia synergise through NF-κB/HIF-1α to upregulate VEGF, bFGF, PDGF, ANGPT2, and PDGFB, while anti-angiogenic factors (TSP-1, angiostatin, endostatin) are downregulated and MMP balance is disturbed. CXCR2^+^ endothelial cells are recruited (IL-18); the result is hyper-dense yet structurally and functionally abnormal vasculature with a self-perpetuating HIF-1α–inflammation feedback loop. **(D)** Pre-metastatic niche formation. Primary-tumour-derived G-CSF, GM-CSF, VEGF, PlGF, S100A8/A9, LOX, and inflammatory cytokines mobilise BMDCs along chemokine gradients to distant organs (lung, liver, bone). TGF-β/PDGF-activated, myofibroblast-like CAFs remodel the ECM via MMPs, degrading basement membrane. VCAM-1 and E-selectin support tumour-cell adhesion and extravasation; VEGF and PlGF maintain vascular permeability; complement C5a– and leukotriene B4 (LTB4)–driven neutrophil recruitment and NET-mediated CTC trapping construct a pre-existing immunosuppressive milieu, facilitating metastatic colonisation. **(E)** Immunosuppressive tumour microenvironment. *MDSC expansion and recruitment:* IL-6, IL-1β, and G-CSF drive bone-marrow proliferation; DAMPs, TGF-β, G-CSF, and GM-CSF together with CCL2, CXCL1/2, and S100A8/A9 traffic MDSCs to the TME, where ARG1, iNOS/NO/ROS, and IL-10/TGF-β suppress T-cell function. *Treg recruitment and stabilisation:* IL-1β/TGF-β drive Foxp3^+^ Treg differentiation; the IDO–kynurenine–AHR axis stabilises Tregs; CCL22/CCL17–CCR4 mediates Treg homing; Tregs in turn suppress effector T cells via TGF-β/IL-10, CTLA-4–CD80/86 engagement, and CD39/CD73-driven adenosine production. *Immune checkpoints:* IFN-γ–JAK–STAT1 upregulates PD-L1 on tumour and myeloid cells; IL-6–STAT3 supports PD-1, TIM-3, LAG-3, and TIGIT. *Metabolic suppression:* tumour-derived lactate and PGE2 lower pH and engage A2A–cAMP–PKA signalling, blunting T- and NK-cell function. Net outcome: immune evasion and resistance to immunotherapy. **(F)** Epithelial–mesenchymal transition and invasive phenotype. IL-1β and TGF-β activate EMT-TFs (Snail, Slug, Twist, ZEB1) through Wnt/β-catenin (TCF/LEF → EMT/CSC genes), Notch (→ Snail; EMT, IL-6, Jagged1), and MAPK/PI3K-AKT pathways. The phenotypic switch downregulates epithelial markers (E-cadherin, claudins, occludin) and upregulates mesenchymal markers (N-cadherin, vimentin, fibronectin). Concurrent ECM remodelling by TGF-β/PDGF-activated, myofibroblast-like CAFs supports a “partial EMT” (pEMT) state with hybrid features and high metastatic seeding and stemness, promoting local invasion, dissemination, and therapy resistance. AHR, aryl hydrocarbon receptor; ALDH1, aldehyde dehydrogenase 1; ANGPT2, angiopoietin 2; APC, adenomatous polyposis coli; ARG1, arginase 1; bFGF, basic fibroblast growth factor; BMDC, bone marrow–derived cell; CAF, cancer-associated fibroblast; CSC, cancer stem cell; CTLA-4, cytotoxic T-lymphocyte–associated protein 4; DNMT, DNA methyltransferase; ECM, extracellular matrix; EMT, epithelial–mesenchymal transition; EMT-TFs, EMT transcription factors; G-CSF/GM-CSF, granulocyte/granulocyte–macrophage colony-stimulating factor; HDAC, histone deacetylase; HIF-1α, hypoxia-inducible factor 1-alpha; IDO, indoleamine 2, 3-dioxygenase; IL, interleukin; iNOS, inducible nitric oxide synthase; LAG-3, lymphocyte-activation gene 3; LGR5, leucine-rich repeat–containing G protein–coupled receptor 5; LOX, lysyl oxidase; LTB4, leukotriene B4; MDSC, myeloid-derived suppressor cell; MMPs, matrix metalloproteinases; NANOG, Nanog homeobox; NF-κB, nuclear factor κB; NK, natural killer cell; OCT4, octamer-binding transcription factor 4; PDCD4, programmed cell death 4; PDGF/PDGFB, platelet-derived growth factor/PDGF-B; pEMT, partial epithelial–mesenchymal transition; PD-1, programmed cell death protein 1; PD-L1, programmed death-ligand 1; PGE2, prostaglandin E2; PI3K-AKT, phosphoinositide 3-kinase–protein kinase B; PlGF, placental growth factor; PTEN, phosphatase and tensin homolog; ROS, reactive oxygen species; SOX2, SRY-box transcription factor 2; STAT, signal transducer and activator of transcription; TAM, tumour-associated macrophage; TCF/LEF, T-cell factor/lymphoid enhancer factor; TGF-β, transforming growth factor beta; TIGIT, T-cell immunoreceptor with Ig and ITIM domains; TIM-3, T-cell immunoglobulin and mucin domain–containing 3; TNF-α, tumour necrosis factor alpha; Treg, regulatory T cell; TSP-1, thrombospondin-1; VCAM-1, vascular cell adhesion molecule 1; VEGF, vascular endothelial growth factor; ZEB1, zinc finger E-box binding homeobox 1. The phrase “vicious cycle” in panel C of the original legend has been replaced with the neutral “feedback loop”, in line with the manuscript-wide effort to reduce stylistic markers flagged by the reviewer. The same change was made in the main text.

**Figure 6** is a six-panel central-radial schematic. The central node depicts pyroptotic TAMs and pyroptotic tumor cells as the shared upstream source of chronic, low-grade IL-1β, IL-18, TNF-α, IL-6, CXCL8, and DAMPs, the “pyroptosis-driven inflammatory storm.” Six surrounding panels (A–F), each corresponding to one Section 4.1 subsection, depict the resulting pro-tumor consequences: (A) genomic instability and epigenetic reprogramming (DNA damage from reactive oxygen and nitrogen species (ROS/RNS), p53/APC mutations, IL-6–STAT3-driven DNA methyltransferase (DNMT) activation and CpG hypermethylation, histone deacetylase (HDAC)–driven oncogene activation, miR-155/miR-21 → phosphatase and tensin homolog (PTEN)/programmed cell death 4 (PDCD4) downregulation) — Section 4.1.1; (B) cancer stem cell enrichment (IL-1β/IL-6/IL-8–driven STAT3, Wnt/β-catenin, Notch, Hedgehog activation; CD44, aldehyde dehydrogenase 1 (ALDH1), leucine-rich repeat–containing G protein–coupled receptor 5 (LGR5), SOX2/OCT4/NANOG upregulation; lower sub-block also depicts vascular permeability changes) — Section 4.1.1 final paragraph; (C) pro-angiogenic remodeling (IL-1β/TNF-α/hypoxia → NF-κB/HIF-1α → VEGF/basic fibroblast growth factor (bFGF)/platelet-derived growth factor (PDGF)/angiopoietin 2 (ANGPT2)/PDGFB; anti-angiogenic factors downregulated; CXCR2-recruited endothelial response; HIF-1α–inflammation feedback loop) — Section 4.1.2; (D) pre-metastatic niche formation (granulocyte colony-stimulating factor (G-CSF)/granulocyte–macrophage colony-stimulating factor (GM-CSF)/VEGF/placental growth factor (PlGF)/S100A8-A9/lysyl oxidase (LOX) → bone marrow–derived cell (BMDC) mobilization; ECM pre-remodeling by TGF-β/PDGF-activated cancer-associated fibroblasts (CAFs) and MMPs; vascular permeability changes; pre-existing immunosuppressive milieu) — Section 4.1.5; (E) immunosuppressive TME (myeloid-derived suppressor cell (MDSC) expansion/recruitment via IL-6/IL-1β/G-CSF and CCL2/CXCL1-2/S100A8-A9; Treg recruitment and stabilization via TGF-β and indoleamine 2, 3-dioxygenase (IDO)–kynurenine–aryl hydrocarbon receptor (AHR); immune checkpoint upregulation; metabolic suppression by lactate/low pH/PGE2 → adenosine A2A receptor (A2A)–cAMP–PKA) — Section 4.1.3; (F) EMT and invasive phenotype (IL-1β/TGF-β-driven EMT transcription factor (EMT-TF) activation via Wnt/β-catenin/Notch/MAPK/phosphoinositide 3-kinase–protein kinase B (PI3K-AKT); epithelial-to-mesenchymal marker switch; CAF activation and ECM remodeling; partial EMT and plasticity) — Section 4.1.4. Throughout Section 4.1, please consult the corresponding panel letter rather than returning to the full figure repeatedly.

#### 4.1.1 Chronic inflammation and tumorigenesis

Approximately 15–20% of malignancies are linked to chronic inflammation or persistent infection, such as *Helicobacter pylori* with gastric cancer, hepatitis B/C with hepatocellular carcinoma, and inflammatory bowel disease (IBD) with colorectal cancer (103). Several interlocking mechanisms have been proposed (**Figure 6A**).

##### 4.1.1.1 Genomic instability

Activated macrophages and neutrophils continuously generate ROS/RNS via nicotinamide adenine dinucleotide phosphate (NADPH) oxidase, iNOS, and myeloperoxidase. Superoxide reacting with NO yields peroxynitrite (ONOO^-^), a potent oxidant that damages DNA directly and impairs DNA repair enzymes (104, 105). The resulting oxidative lesions, including 8-hydroxy-2′-deoxyguanosine (8-OHdG) and single- and double-strand breaks, accumulate in chronically inflamed tissue, with γ-H2A histone family member X (γH2AX)–positive foci providing an *in situ* readout. IL-1β and TNF-α reinforce this through NF-κB–driven iNOS/NADPH oxidase (NOX) upregulation, while concurrent down-regulation of base-excision and mismatch-repair pathways may compound the mutational burden (106). This “increased damage – decreased repair” pattern is consistent with the elevated cancer incidence observed in chronically inflamed tissues (see **Figure 6A**).

##### 4.1.1.2 Epigenetic reprogramming

IL-6/STAT3 signaling has been linked to DNMT activation and CpG-island hypermethylation of tumor-suppressor promoters (107), while inflammation-associated HDAC activity may shift the histone-modification landscape at oncogene loci such as *MYC* and *CCND1* (108). Inflammation-induced microRNAs are also implicated: miR-155 is a direct NF-κB target, and miR-21 represses tumor suppressors including *PTEN* and *PDCD4* (109). Because epigenetic changes are reversible, HDAC and DNMT inhibitors remain plausible candidates for prevention strategies in inflammation-associated cancers (see **Figure 6A**, lower section).

##### 4.1.1.3 Cancer stem cell enrichment

Inflammatory cytokines (IL-6, IL-8, IL-1β) and hypoxia jointly activate stemness pathways (Wnt/β-catenin, Notch, Hedgehog) via STAT3 and NF-κB/HIF-1α signaling, and have been associated with up-regulation of cancer stem cell (CSC) markers (CD44, ALDH1, LGR5) and pluripotency factors (SOX2, OCT4, NANOG) (**Figure 6B**) (110). CSCs may contribute to chemo- and radioresistance and to tumor relapse (111). Targeting the inflammation–CSC axis is therefore an attractive strategy to address resistance (see **Figure 6B**).

#### 4.1.2 Promotion of angiogenesis

Inflammatory storms can promote tumor neovascularization through several converging mechanisms (see **Figure 6C**) (112). IL-1β and TNF-α act synergistically through NF-κB and HIF-1α to up-regulate VEGF, bFGF, PDGF, and ANGPT2; CXCL8 also signals directly through CXCR1/2 to promote endothelial migration and tube formation (113, 114). Even under normoxia, ROS, NO, and inflammatory metabolites can inhibit prolyl hydroxylases and stabilize HIF-1α, broadening the transcriptional output of pro-angiogenic genes (13). In parallel, endogenous angiogenesis brakes such as thrombospondin-1 (TSP-1) are down-regulated (115). The result is hyper-dense but structurally and functionally abnormal vasculature: tortuous, leaky, with discontinuous basement membrane and poor pericyte coverage (91). Vascular normalization therapy, which moderately curbs angiogenesis to restore perfusion, has been reported to improve chemotherapy and immunotherapy delivery (92, 116).

#### 4.1.3 Formation of an immunosuppressive microenvironment

Sustained inflammation paradoxically generates a deeply immunosuppressive TME, arguably the clearest manifestation of the pyroptosis “double-edged sword”.

##### 4.1.3.1 Myeloid-derived suppressor cells

IL-6, IL-1β, G-CSF, and GM-CSF expand granulocytic and monocytic MDSC populations from bone marrow and recruit them to tumors via CCL2, CXCL1/2, and S100A8/A9 (117). MDSCs may suppress T cells through arginase-1-driven L-arginine depletion, iNOS/NO/ROS-mediated T-cell receptor (TCR) damage, and IL-10/TGF-β secretion (118). CCR2 blockade combined with chemotherapy has shown promising efficacy in early clinical studies (see **Figure 6E**, MDSC sub-block) (119).

##### 4.1.3.2 Regulatory T cells (Tregs)

TGF-β promotes Foxp3^+^ Treg differentiation, while inflammation-induced IDO activity generates kynurenine that further stabilizes Tregs through AHR signaling (120). CCL22/CCL17–CCR4 chemokines recruit Tregs to tumors, where they suppress effector function via TGF-β and IL-10 secretion, CTLA-4 engagement of CD80/CD86, and CD39/CD73–driven adenosine production (121). Tumor-infiltrating Treg subsets with enhanced suppressive capacity have been described and may be more selective therapeutic targets than the bulk Treg population (see **Figure 6E**, Treg sub-block) (122).

##### 4.1.3.3 Checkpoints and metabolic immunosuppression

IFN-γ paradoxically up-regulates PD-L1 on tumor and myeloid cells through JAK/STAT1 (123), while IL-6/STAT3 and chronic activation drive PD-1, TIM-3, LAG-3, and T-cell immunoreceptor with Ig and ITIM domains (TIGIT) expression on T cells. Local lactate accumulation, low pH, and elevated PGE2 further blunt T and NK cell function via A2A–cAMP–PKA signaling (124). These layered mechanisms cooperate to construct the immunosuppressive network and provide a rationale for combination therapies (see **Figure 6E**, checkpoint sub-block).

Beyond the cytokine-driven mechanisms described above, TAMs can also act as a structural barrier to checkpoint blockade by physically constraining T-cell access to tumor islets and by sustaining a metabolite-rich suppressive milieu, a view recently consolidated in the context of immunotherapy resistance (11). The chronic, low-grade cytokine signature emerging from sub-lytic TAM pyroptosis described in Section 4.1.1 may represent one upstream driver of this barrier function.

#### 4.1.4 Induction of epithelial–mesenchymal transition

Inflammatory mediators are potent EMT inducers, conferring mesenchymal, invasive, and stem-like properties on tumor cells (**Figure 6F**). TGF-β, the most potent EMT-inducing cytokine, activates EMT-TFs (Snail, Slug, Twist, ZEB1) through small mothers against decapentaplegic (SMAD)–dependent and –independent pathways (125), suppressing epithelial markers (E-cadherin, claudins) and inducing mesenchymal markers (N-cadherin, vimentin). IL-1β contributes via NF-κB-dependent Snail induction (126), while IL-6 and Wnt/β-catenin signaling provide additional, partly redundant inputs (127). In parallel, TGF-β and PDGF activate CAFs, which remodel the ECM and release matrix-bound growth factors via MMPs (see **Figure 6F**) (128, 129).

Recent single-cell studies indicate that EMT is not a binary switch but a continuum, with hybrid epithelial/mesenchymal partial EMT (pEMT) cells appearing to possess the highest metastatic and stem-like potential (130). This framing has refined the traditional EMT model and may help explain why blocking any single EMT-TF is unlikely to fully suppress metastasis.

#### 4.1.5 Shaping of the pre-metastatic niche

The systemic effects of pyroptotic inflammation may extend well beyond the primary tumor, contributing to “pre-metastatic niches” in distant organs. Primary-tumor–derived G-CSF, GM-CSF, VEGF, PlGF, and S100A8/A9 mobilize vascular endothelial growth factor receptor 1 (VEGFR1)^+^ BMDCs that home to specific organs and remodel the local microenvironment (131, 132). LOX released from primary tumors cross-links collagen at distant sites, generating a fibrotic scaffold permissive for metastatic colonization; LOX inhibition reduces metastasis in preclinical models (see **Figure 6D**) (133). VEGF and inflammatory mediators increase niche vascular permeability, providing extravasation entry points for circulating tumor cells (134), while early MDSC, Treg, and M2-like macrophage infiltration establishes immunosuppressive cover (135). Differential responsiveness of organ microenvironments may also help explain the well-documented organotropism of metastasis (136).

### 4.2 Anti-tumor effects

Cell-type scope note: anti-tumor effects below involve contributions from both TAM pyroptosis (IL-1β, IL-18, IFN-I, chemokines) and tumor-cell pyroptosis (antigens, calreticulin, HMGB1).

**Figure 7:**
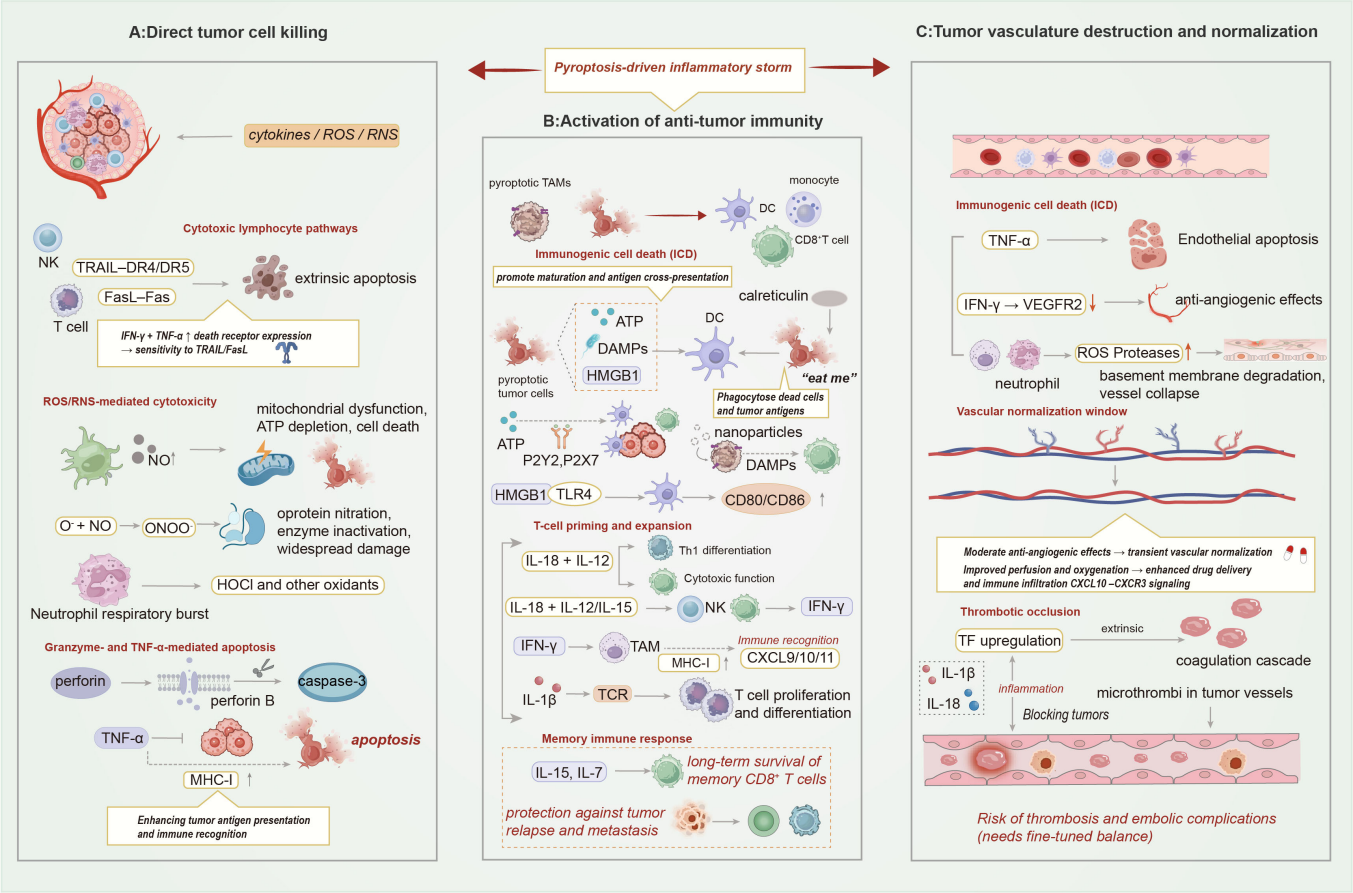
Acute pyroptotic inflammation drives three anti-tumor effects. The central banner depicts the pyroptosis-driven inflammatory storm as the shared upstream source. Three panels show the principal acute anti-tumour consequences. **(A)** Direct tumour cell killing. Cytotoxic effectors converge on multiple death modalities: NK-cell– and CTL-derived TRAIL → DR4/DR5 and FasL → Fas drive extrinsic apoptosis, with IFN-γ + TNF-α further upregulating death receptor expression and tumour-cell sensitivity. ROS/RNS-mediated cytotoxicity (NO → mitochondrial dysfunction, ATP depletion; superoxide + NO → ONOO^-^ → protein nitration, enzyme inactivation; HOCl from neutrophil respiratory burst) damages tumour cells. Perforin enables granzyme B entry to activate caspase-3–mediated apoptosis; TNF-α contributes via TNFR1 death signalling; concomitant MHC-I upregulation enhances tumour antigen presentation and immune recognition. **(B)** Activation of anti-tumour immunity. Pyroptotic tumour cells and TAMs release ATP, HMGB1, and other DAMPs, and expose calreticulin (CRT) as an “eat-me” signal; DCs phagocytose dead cells and tumour antigens, mature (HMGB1 → TLR4 → CD80/CD86 upregulation), and cross-present antigens to CD8^+^ T cells. ATP–P2Y2/P2X7 supports DC recruitment/activation. T-cell priming and expansion proceed through several axes: IL-18 + IL-12 → Th1 differentiation and cytotoxic function; IL-18 + IL-12/IL-15 → NK-cell IFN-γ; IFN-γ → TAM MHC-I upregulation and CXCL9/10/11 induction (immune recognition); IL-1β engages the TCR signalling axis as a “third signal” supporting T-cell proliferation and effector differentiation. The lower dashed box highlights memory-immunity formation: IL-15/IL-7 sustain memory CD8^+^ T cells, providing protection against tumour relapse and metastasis. **(C)** Tumour vasculature destruction and normalisation. ICD-derived TNF-α and IFN-γ trigger endothelial apoptosis and downregulate VEGFR2, generating anti-angiogenic effects; neutrophil-derived ROS and proteases degrade basement membrane and collapse vessels. In the moderate intensity range, these effects open a transient “vascular normalisation window” that improves perfusion, oxygenation, drug delivery, and immune infiltration, partly mediated by CXCL10/CXCR3 signalling. At higher intensity, IL-1β/IL-18-driven inflammation upregulates tissue factor (TF), engaging the extrinsic coagulation cascade and producing microthrombi, blocking tumour blood supply. The same mechanism, however, raises the risk of thromboembolic complications, requiring fine-tuned balance. Colour code: red text = key biological outcome; dashed box = highlighted mechanism (memory immunity in B; vascular normalisation window in C). ATP, adenosine triphosphate; CD80/CD86, B7-1/B7–2 co-stimulatory molecules; CRT, calreticulin; CTL, cytotoxic T lymphocyte; CXCL, C-X-C motif chemokine ligand; CXCR3, C-X-C motif chemokine receptor 3; DAMPs, damage-associated molecular patterns; DC, dendritic cell; DR4/DR5, death receptor 4/5; FasL, Fas ligand; HMGB1, high-mobility group box 1; HOCl, hypochlorous acid; ICD, immunogenic cell death; IFN-γ, interferon gamma; IL, interleukin; MHC-I, major histocompatibility complex class I; NK, natural killer cell; NO, nitric oxide; ONOO^-^, peroxynitrite; P2X7/P2Y2, purinergic receptors P2X 7/P2Y 2; RNS, reactive nitrogen species; ROS, reactive oxygen species; TAM, tumour-associated macrophage; TCR, T-cell receptor; TF, tissue factor; TLR4, Toll-like receptor 4; TNF-α/TNFR1, tumour necrosis factor alpha/TNF receptor 1; TRAIL, TNF-related apoptosis-inducing ligand; VEGFR2, vascular endothelial growth factor receptor 2.

Although chronic, low-grade inflammation tends to favor tumor progression, *acute*, high-amplitude pyroptotic inflammation can drive substantial anti-tumor effects (**Figure 7**). These contributions come from both TAM pyroptosis (providing IL-1β, IL-18, IFN-I, and chemokines that license effector lymphocytes) and tumor-cell pyroptosis (providing tumor antigens, calreticulin (CRT), and HMGB1 for DC activation).

#### 4.2.1 Direct tumor cell killing

Pyroptotic macrophages, NK cells, and CD8^+^ T cells release cytotoxic effectors that engage multiple death modalities (**Figure 7A**). NK and CTL granule contents (perforin, granzyme B) trigger caspase-mediated apoptosis, while TNF-related apoptosis-inducing ligand (TRAIL) engaging death receptor 4/5 (DR4/DR5) and Fas ligand (FasL) engaging Fas (CD95) activate the extrinsic apoptotic pathway with relative tumor-cell selectivity due to differential decoy-receptor expression on normal cells (137). TNF-α at sufficient local concentrations drives TNFR1 death signaling (77), and IFN-γ has both direct anti-proliferative and indirect (major histocompatibility complex class I (MHC-I) up-regulation, antigen presentation enhancement) effects (138).

Acute inflammation also generates high local concentrations of ROS and RNS, including ONOO^-^ and hypochlorous acid (HOCl), which can produce mitochondrial dysfunction, ATP depletion, and protein nitration in tumor cells (139). These effects are concentration-dependent: at low chronic levels the same mediators may instead promote tumor survival, which underlies the divergent outcomes of chronic versus acute inflammation. A caveat is that ROS/RNS-mediated cytotoxicity lacks intrinsic specificity, so targeted delivery remains a key technical challenge.

#### 4.2.2 Activation of anti-tumor immunity

Pyroptosis is regarded as a highly immunogenic form of cell death, in contrast to apoptosis, which is comparatively immunologically silent (**Figure 7B**). Pyroptotic tumor cells release CRT (“eat-me” signal), ATP (signaling through P2Y2/P2X7 to recruit and activate DCs), and HMGB1 (signaling through TLR4 to promote DC maturation) (87). Mature DCs up-regulate CD80/CD86 and cross-present tumor antigens, priming and expanding tumor-specific CD8^+^ T cells.

The bioorthogonal study in the murine 4T1 model reported that pyroptosis induced in fewer than 15% of tumor cells was sufficient to clear tumor grafts in a T-cell–dependent manner, accompanied by enhanced anti–PD-1 efficacy (43). *Caveat:* this finding derives from a single model and warrants replication in additional tumor types before generalization. The data nonetheless support the idea that pyroptosis can act as an “endogenous vaccine” capable of converting “cold” tumors to “hot”.

Subsequent T-cell priming is shaped by IL-12 and IL-18 (Th1 differentiation), IL-15 (NK and memory CD8^+^ T cell support), and IFN-γ-induced CXCL9/10/11, which recruit additional effector lymphocytes via CXCR3 (140). IL-1β has been described as the “third signal” for full T-cell effector differentiation; T cells lacking this signal can be activated but show impaired effector function and memory formation, indicating that some level of inflammation may be required, not merely tolerated, for durable immunity (141).

#### 4.2.3 Disruption of tumor vasculature

Acute inflammatory mediators can also damage tumor vasculature (**Figure 7C**). TNF-α and IFN-γ released during immunogenic cell death can induce endothelial apoptosis, while neutrophil-derived ROS and proteases degrade the basement membrane, leading to vessel collapse (142). Historically, high-dose systemic TNF-α was limited by toxicity, motivating local or tumor-targeted delivery strategies. *Moderate* anti-angiogenic effects can transiently normalize tumor vasculature, transiently improving perfusion, drug delivery, and immune cell infiltration, a therapeutic window potentially modulated by CXCL10/CXCR3 signaling (143). Inflammation-driven tissue factor expression can also seed microthrombi that cut off tumor blood supply, though this same mechanism contributes to thromboembolic risk and demands careful clinical balancing (144).

### 4.3 Determinants of outcome

Whether a pyroptosis-driven cytokine storm favors tumor progression or regression appears to depend on several modifiable and non-modifiable factors. Tumor-intrinsic features matter: inflammation-driven cancers (e.g., hepatocellular carcinoma, gastric cancer) are particularly dependent on IL-6/STAT3 signaling, with IL-6 inhibition showing efficacy in selected contexts (145), while microsatellite-instability-high (MSI-H) tumors with high mutational burden are more readily recognized by pyroptosis-activated immunity (146). Disease stage also matters; early-stage tumors with relatively intact immune surveillance respond more reliably to acute inflammatory induction than do advanced tumors that have undergone immunoediting (147).

Intensity and temporal pattern are also relevant. The relationship between inflammation magnitude and tumor outcome is non-linear, with both insufficient and excessive responses associated with poor results (148); landing precisely within a “therapeutic window” remains a major challenge and motivates the development of biomarker-guided dosing. Acute, pulsatile inflammation tends to support anti-tumor immunity, whereas chronic exposure tends to entrench an immunosuppressive niche (149), a kinetic principle now being explored in immunotherapy regimen design.

Microenvironmental and host context further modulate outcome. Baseline immune fitness predicts response (150); tumor metabolic features (lactate, low pH) blunt effector function (100); and ECM stiffness and architecture shape immune cell infiltration (100). At the systemic level, comorbidities, nutritional status, and age-associated “inflammaging, “ meaning chronically elevated baseline inflammation coupled with reduced response capacity, shift the dose–response curve and warrant individualized assessment in elderly patients (151). The framing of pyroptosis as a context-dependent double-edged effector also applies outside oncology, including in sterile and infectious inflammatory diseases (152); the present review is restricted to the oncological context and to the macrophage compartment.

## 5 Clinical relevance

Macrophage pyroptosis and cytokine storm biology have moved beyond mechanistic interest into clinically translatable territory. Understanding how these processes manifest across tumor types, identifying robust biomarkers, and assessing prognostic value are increasingly relevant for individualized diagnosis and treatment.

### 5.1 Manifestations in different tumor types

#### 5.1.1 Solid tumors

##### 5.1.1.1 Lung cancer

GSDMD protein is reportedly upregulated in non-small cell lung cancer (NSCLC), where high expression has been associated with larger tumors, more advanced tumor–node–metastasis (TNM) stage, and, in lung adenocarcinoma but not lung squamous cell carcinoma, poorer prognosis (153). GSDMD knockdown appears to suppress tumor proliferation in part through inhibition of epidermal growth factor receptor (EGFR)/AKT signaling. GSDME is often *down*-regulated in lung tumors and has been proposed as a tumor suppressor whose expression supports anti-tumor immunity by enabling cytotoxic-lymphocyte–induced tumor pyroptosis (4); this raises the possibility that GSDME status could help guide chemotherapy selection.

##### 5.1.1.2 Hepatocellular carcinoma

As a prototypical inflammation-associated cancer, HCC illustrates the NLRP3 “double-edged sword”: chronic IL-1β/IL-18 signaling supports tumorigenesis, while acute NLRP3 activation can purge damaged hepatocytes (154). Pan-cancer single-cell transcriptomics has resolved a heterogeneous tumor-infiltrating myeloid landscape in HCC with distinct functional subsets (34). Sorafenib has been shown to induce macrophage pyroptosis and trigger NK-cell–mediated cytotoxicity, with the anti-tumor effect substantially attenuated when IL-1β and IL-18 are blocked *in vivo* (155).

##### 5.1.1.3 Colorectal cancer

Pyroptosis-related gene signatures stratify CRC patients into prognostic subgroups, with low-score tumors showing stronger immune infiltration and possible immunotherapy benefit (156). The clearest clinical application is in mismatch-repair–deficient/microsatellite-instability-high (dMMR/MSI-H) metastatic CRC, where nivolumab plus ipilimumab produces durable benefit (157), suggesting that local immune activation strategies may be particularly valuable in this subset. Strategies that re-polarize TAMs from M2- toward M1-like states, for example oncolytic adenovirus engineered to express neutrophil elastase/proteinase 3 to enhance effector memory CD8^+^ T cell infiltration, have been reported to augment anti-CRC efficacy.

##### 5.1.1.4 Pancreatic ductal adenocarcinoma

PDAC is characterized by dense stroma and a “cold” immune phenotype. Some chemotherapeutic agents activate caspase-3 to cleave GSDME, converting apoptosis to pyroptosis in tumor-cell subsets and potentially augmenting immunogenicity (16). Pseudoprogression, apparent radiographic enlargement reflecting immune infiltration rather than tumor growth, can complicate response assessment with immunotherapy across solid tumors and warrants careful clinical interpretation (158).

#### 5.1.2 Hematological malignancies

##### 5.1.2.1 Acute myeloid leukemia

NLRP3/pyroptosis biology in AML is context-dependent and has been described as either pro- or anti-tumor (159). Chemotherapy-induced leukemia-cell pyroptosis releases DAMPs that may license dendritic-cell–mediated antigen presentation, but excessive pyroptosis is also implicated in severe post-chemotherapy complications.

##### 5.1.2.2 Multiple myeloma

Bone-marrow microenvironment IL-18 has been described as a key driver of immunosuppression in MM, sustaining MDSC populations; high bone-marrow plasma IL-18 has been linked to poorer overall survival (160).

##### 5.1.2.3 CAR-T–associated cytokine release syndrome

Across hematological malignancies treated with CAR-T cells, CRS remains the dose-limiting toxicity. Severe CRS is characterized by hemodynamic instability, capillary leak, and consumptive coagulopathy, with elevated endothelial-activation markers (angiopoietin-2 (Ang-2), von Willebrand factor (vWF)) detectable during the syndrome (44).

##### 5.1.2.4 Lymphoma

Pyroptotic and inflammatory contributions appear subtype-dependent. In diffuse large B-cell lymphoma (DLBCL), the abundance and phenotype of TAMs have been linked to outcome (161).

### 5.2 Biomarker development

#### 5.2.1 Pyroptosis-related protein detection

##### 5.2.1.1 Tissue markers

The N-terminal cleavage fragment of GSDMD (GSDMD-NT) executes pyroptosis by forming membrane pores and is a relatively specific *in situ* marker of pyroptotic activation (162). ASC specks, perinuclear macromolecular inflammasome aggregates, provide an additional histological readout, detectable in tissue sections via fluorescent reporter systems (163).

##### 5.2.1.2 Circulating markers

Soluble GSDMD has been proposed as a candidate non-invasive biomarker in infectious, inflammatory, and oncologic settings (164). cfDNA derives from multiple modes of cell death; its fragmentation pattern carries information about cellular origin and death modality and may complement protein-based markers in the future (165).

#### 5.2.2 Cytokine profiles and composite scores

Single-cytokine measurements have limited predictive value, and multi-analyte panels, typically including IL-6, IFN-γ, and IL-10, generally outperform individual markers in predicting immunotherapy response (166). The IL-1β/IL-18 ratio has also been examined, since IL-18 has context-dependent effects in cancer and free versus IL-18BP-bound IL-18 may carry different biological information (52). Composite scores that integrate systemic inflammation parameters, for example the modified Glasgow Prognostic Score (mGPS) based on C-reactive protein (CRP) and albumin, have been validated as outcome predictors across multiple solid tumors (167). Beyond cancer-specific scores, composite indices originally developed for sepsis, such as the lymphocyte–INR–procalcitonin (LIP) score combining lymphocyte count, international normalized ratio (INR), and procalcitonin, illustrate how multi-parameter inflammatory biomarkers can outperform single markers in predicting systemic inflammatory deterioration, and may be adapted for monitoring CAR-T–associated CRS or pyroptosis-driven cytokine storms.

### 5.3 Prognostic assessment

#### 5.3.1 Overall survival

The prognostic value of pyroptosis-related markers appears to depend on tumor immunogenicity. In immunologically responsive tumors, pyroptosis activation may favor anti-tumor immunity; in tumors embedded in immunosuppressive niches, the same machinery may instead contribute to chronic inflammation and progression (56). GSDME status illustrates this duality, with high tumor GSDME expression linked to enhanced phagocytosis by TAMs and to increased intratumoral NK and CD8^+^ T-cell activity (4).

#### 5.3.2 Treatment response

GSDME expression may also help predict chemotherapy responsiveness: GSDME-high tumors appear more likely to undergo immunogenic pyroptosis on chemotherapy, while GSDME-low tumors default to less immunogenic apoptosis (16). This raises the possibility of selecting regimens by GSDME status, or of using demethylating agents to restore GSDME expression in silenced tumors.

#### 5.3.3 Recurrence risk

Persistent peri-tumoral inflammation is thought to support survival of minimal residual disease (MRD) after resection, consistent with the broader role of chronic inflammation in tumor relapse (103). Emerging *in situ* immune-activation strategies, such as oncolytic virus delivery immediately after tumor resection, have been proposed to convert the post-operative inflammatory milieu from pro-tumorigenic to anti-tumorigenic, reducing recurrence in preclinical models.

#### 5.3.4 Adverse event prediction

In CAR-T therapy, severe CRS risk is associated with high pre-treatment tumor burden, low platelet count, the lymphodepletion regimen used (cyclophosphamide/fludarabine), and high CAR-T cell dose (44). Identifying high-risk patients prospectively allows enhanced monitoring and earlier intervention.

## 6 Perspectives

This review has examined the dual roles of macrophage pyroptosis-driven cytokine storms in tumor progression: from the molecular execution of pyroptosis (canonical NLRP3/caspase-1 and non-canonical caspase-4/5/11 pathways converging on GSDMD pore formation), through cascade amplification and signaling networks, to clinical correlates (1, 20). The picture that emerges is consistent with a “double-edged sword”: acute, moderate inflammatory bursts may license anti-tumor immunity through immunogenic cell death (ICD) and effector lymphocyte priming (87), while chronic, low-grade inflammation tends to support angiogenesis, EMT, immunosuppression, and pre-metastatic niche formation (42, 56). Direction and magnitude appear to depend on intensity, duration, spatial scale, and microenvironmental context.

### 6.1 Limitations of current evidence

Several caveats temper extrapolation. Most data come from *in vitro* systems and murine models that may inadequately recapitulate human TME complexity, with documented inter-species differences in inflammasome biology and immune architecture (7). Quantitative relationships between pyroptosis level and downstream cytokine-storm magnitude are poorly defined; standardized assays for *in vivo* pyroptosis intensity are lacking; and the proposed “threshold” proportion of pyroptotic cells needed to ignite a self-amplifying cascade, although informative in single murine models (45), has not been validated across tumor types. Spatial transcriptomics has begun to reveal the heterogeneous geography of intratumoral pyroptosis (43), but tools for tracking cytokine-storm dynamics in real time in patients remain limited. The safety window for therapeutically induced pyroptosis, sufficient to drive immunity without precipitating systemic toxicity, has yet to be defined.

### 6.2 Priority directions

Several lines of work appear well positioned to address these gaps. *First*, precise spatiotemporal control of pyroptosis induction, through nano-delivery, bioorthogonal chemistry, or stimulus-responsive prodrugs, could allow tumor-restricted activation; the bioorthogonal 4T1 result indicating that <15% pyroptotic tumor cells suffice to clear grafts (45) is an encouraging proof of principle, but priority should be replication across additional models, including patient-derived xenografts (PDXs) and humanized mice. *Second*, multiparametric mapping of pyroptotic and cytokine-storm dynamics, combining single-cell multi-omics, intravital imaging, and computational modeling, may help define critical trigger thresholds and regulatory nodes (34). *Third*, rational combination strategies (e.g., chemotherapy- or granzyme B–mediated GSDME activation (4) paired with checkpoint blockade) deserve systematic evaluation. *Fourth*, pyroptosis- and cytokine-based composite biomarkers, incorporating GSDMD/GSDME status, circulating cytokine panels, and immune-infiltrate features, could refine patient selection and toxicity prediction (145). *Fifth*, modulation of endogenous brake systems, including the cGAS–STING axis (168), may help shape both the magnitude and the resolution kinetics of the response.

### 6.3 Translational outlook

Several inflammation-modulating agents have established the feasibility of intervening in this biology. The CANTOS trial linked IL-1β blockade with canakinumab to substantially reduced lung cancer incidence and mortality (49), and IL-6 receptor blockade with tocilizumab is now standard for severe CAR-T cell therapy–associated CRS (15, 169). Pyroptosis- and inflammasome-directed agents, including NLRP3 inhibitors (e.g., MCC950 (CRID3) analogs), caspase-1 inhibitors, and IL-1β/IL-18 blockers, are advancing through early-phase trials (107, 170). Therapeutic intent should, however, be tailored to tumor immunophenotype: “cold” tumors may benefit from pyroptosis induction to ignite immunity, whereas tumors already chronically inflamed may instead require selective dampening.

Recent translational reviews echo this call for context-aware deployment of pyroptosis-inducing strategies, emphasising that the same intervention can produce opposite effects depending on tumor subtype, prior immune status, and the timing of administration (171). Decisions on whether to induce or dampen pyroptosis should therefore be informed by integrated readouts of TAM state, prior IFN exposure, and tumor immunogenicity rather than by GSDMD/GSDME status alone.

## 7 Conclusions

Macrophage pyroptosis-driven cytokine storms exert dual, context-dependent effects on tumor progression, with outcome shaped by the temporal pattern (acute vs chronic), intensity, spatial scale, and immune background of the response. Realising this biology therapeutically will require quantitative tools to titrate pyroptosis induction, spatially controlled delivery technologies, predictive multiparametric biomarkers, and well-designed combinations with established modalities (162, 172). The aspiration of a “controllable cytokine storm”, sufficient to mobilise anti-tumor immunity without precipitating excessive damage, appears achievable, though substantial work remains. Continued progress in single-cell and spatial omics, intravital imaging, and AI-enabled integration is likely to translate these mechanistic insights into a more precise and inflammation-aware era of cancer immunotherapy (173, 174).
