# Supplementary material for: A double-edged sword: the role of macrophage pyroptosis-driven cytokine storm-mediated intercellular communication in tumor progression
Source: Front Immunol. 2026 Jul 21;17:1828486. doi: 10.3389/fimmu.2026.1828486 (PMC13433866; doi:10.3389/fimmu.2026.1828486)
Supplement: Supplementary file 1 [file Table1.docx]

**List of Abbreviations**

| **Abbreviation** | **Full term** |
| --- | --- |
| **8-OHdG** | 8-hydroxy-2′-deoxyguanosine |
| **A20** | TNF-α–induced protein 3 (TNFAIP3) |
| **A2A** | adenosine A2A receptor |
| **AhR** | aryl hydrocarbon receptor |
| **AIM2** | absent in melanoma 2 |
| **ALDH1** | aldehyde dehydrogenase 1 |
| **AML** | acute myeloid leukaemia |
| **Ang-2** | angiopoietin 2 |
| **ANGPT2** | angiopoietin 2 |
| **AP-1** | activator protein 1 |
| **APC** | adenomatous polyposis coli |
| **Arg1** | arginase 1 |
| **ASC** | apoptosis-associated speck-like protein containing a CARD |
| **ATP** | adenosine triphosphate |
| **BCG** | Bacillus Calmette–Guérin |
| **bFGF** | basic fibroblast growth factor |
| **BMDC** | bone marrow–derived cell |
| **CAF** | cancer-associated fibroblast |
| **CANTOS** | Canakinumab Anti-inflammatory Thrombosis Outcome Study |
| **CAR-T** | chimeric antigen receptor T-cell |
| **CARD** | caspase activation and recruitment domain |
| **CCL** | C-C motif chemokine ligand |
| **CCR** | C-C motif chemokine receptor |
| **CD39** | ectonucleoside triphosphate diphosphohydrolase 1 |
| **CD73** | ecto-5′-nucleotidase |
| **cDC1** | type 1 conventional dendritic cell |
| **cfDNA** | cell-free DNA |
| **cGAS–STING** | cyclic GMP–AMP synthase–stimulator of interferon genes |
| **circRNA** | circular RNA |
| **CNS** | central nervous system |
| **COX-2** | cyclooxygenase-2 |
| **CpG** | cytosine–phosphate–guanine |
| **CRC** | colorectal cancer |
| **CRID3** | MCC950 (NLRP3 inhibitor) |
| **CRP** | C-reactive protein |
| **CRS** | cytokine release syndrome |
| **CRT** | calreticulin |
| **CSC** | cancer stem cell |
| **CSF-1** | colony-stimulating factor 1 |
| **CTC** | circulating tumour cell |
| **CTL** | cytotoxic T lymphocyte |
| **CTLA-4** | cytotoxic T-lymphocyte–associated protein 4 |
| **CXCL** | C-X-C motif chemokine ligand |
| **CXCR** | C-X-C motif chemokine receptor |
| **DAMP** | damage-associated molecular pattern |
| **DC** | dendritic cell |
| **DFNA5** | deafness, autosomal dominant 5 (synonym for GSDME) |
| **DIC** | disseminated intravascular coagulation |
| **DLBCL** | diffuse large B-cell lymphoma |
| **dMMR** | mismatch-repair–deficient |
| **DMB** | 3,3-dimethyl-1-butanol |
| **DNMT** | DNA methyltransferase |
| **DR4 / DR5** | death receptor 4 / 5 |
| **dsRNA** | double-stranded RNA |
| **ECM** | extracellular matrix |
| **EGFR** | epidermal growth factor receptor |
| **ELISA** | enzyme-linked immunosorbent assay |
| **EMT** | epithelial–mesenchymal transition |
| **EMT-TF** | EMT transcription factor |
| **ESCRT-III** | endosomal sorting complex required for transport III |
| **FAO** | fatty acid oxidation |
| **FasL** | Fas ligand |
| **FDA** | U.S. Food and Drug Administration |
| **FFAR2 / FFAR3** | free fatty acid receptor 2 / 3 |
| **FGF-2** | fibroblast growth factor 2 |
| **FLICA** | fluorescent-labelled inhibitor of caspases |
| **G-CSF** | granulocyte colony-stimulating factor |
| **GBP** | guanylate-binding protein |
| **GM-CSF** | granulocyte–macrophage colony-stimulating factor |
| **gp130** | glycoprotein 130 |
| **gp96** | glycoprotein 96 |
| **GPCR** | G protein–coupled receptor |
| **GPR41 / 43 / 81 / 109A** | G protein–coupled receptor 41 / 43 / 81 / 109A |
| **GSDMD** | gasdermin D |
| **GSDMD-NT** | GSDMD N-terminal fragment |
| **GSDME** | gasdermin E |
| **HCC** | hepatocellular carcinoma |
| **HDAC** | histone deacetylase |
| **HIF-1α** | hypoxia-inducible factor 1-alpha |
| **HIN200** | hematopoietic interferon-inducible nuclear protein with a 200-amino-acid repeat |
| **HLH / MAS** | haemophagocytic lymphohistiocytosis / macrophage activation syndrome |
| **HMGB1** | high-mobility group box 1 |
| **HOCl** | hypochlorous acid |
| **HPA** | hypothalamic–pituitary–adrenal axis |
| **HSP** | heat shock protein |
| **IBD** | inflammatory bowel disease |
| **ICAM-1** | intercellular adhesion molecule 1 |
| **ICD** | immunogenic cell death |
| **ICI** | immune checkpoint inhibitor |
| **IDO** | indoleamine 2,3-dioxygenase |
| **IFN** | interferon |
| **IFNAR** | type I interferon receptor |
| **IFN-I** | type I interferon |
| **IFN-γ** | interferon gamma |
| **IL** | interleukin |
| **IL-18BP** | IL-18 binding protein |
| **IL-1R1** | IL-1 receptor type 1 |
| **IL-1Ra** | IL-1 receptor antagonist |
| **iNOS** | inducible nitric oxide synthase |
| **INR** | international normalised ratio |
| **IRAK** | IL-1 receptor–associated kinase |
| **IRF** | interferon regulatory factor |
| **ISG** | interferon-stimulated gene |
| **JAK** | Janus kinase |
| **LAG-3** | lymphocyte-activation gene 3 |
| **LDH** | lactate dehydrogenase |
| **LGR5** | leucine-rich repeat–containing G protein–coupled receptor 5 |
| **LIP** | lymphocyte–INR–procalcitonin score |
| **lncRNA** | long non-coding RNA |
| **LOX** | lysyl oxidase |
| **LPS** | lipopolysaccharide |
| **LRR** | leucine-rich repeat |
| **LTB4** | leukotriene B4 |
| **MAPK** | mitogen-activated protein kinase |
| **MCC950** | diarylsulfonylurea NLRP3 inhibitor (synonym CRID3) |
| **MDA5** | melanoma differentiation–associated protein 5 |
| **MDSC** | myeloid-derived suppressor cell |
| **mGPS** | modified Glasgow Prognostic Score |
| **MHC-I / II** | major histocompatibility complex class I / II |
| **miRNA / miR** | microRNA |
| **MM** | multiple myeloma |
| **MMP** | matrix metalloproteinase |
| **MRD** | minimal residual disease |
| **MSI-H** | microsatellite-instability-high |
| **mtDNA** | mitochondrial DNA |
| **MyD88** | myeloid differentiation primary response 88 |
| **NACHT** | NAIP–CIITA–HET-E–TP1 domain |
| **NADPH** | nicotinamide adenine dinucleotide phosphate |
| **ncRNA** | non-coding RNA |
| **NET** | neutrophil extracellular trap |
| **NETosis** | neutrophil extracellular trap formation |
| **NF-κB** | nuclear factor kappa-light-chain-enhancer of activated B cells |
| **NINJ1** | ninjurin-1 |
| **NK** | natural killer (cell) |
| **NLRC4** | NLR family CARD domain–containing 4 |
| **NLRP3** | NLR family pyrin domain–containing 3 |
| **NO** | nitric oxide |
| **NSCLC** | non-small cell lung cancer |
| **ONOO⁻** | peroxynitrite |
| **ox-mtDNA** | oxidised mitochondrial DNA |
| **OXPHOS** | oxidative phosphorylation |
| **P2X7 / P2Y2** | purinergic receptor P2X 7 / P2Y 2 |
| **PAGln** | phenylacetylglutamine |
| **PAMP** | pathogen-associated molecular pattern |
| **PDAC** | pancreatic ductal adenocarcinoma |
| **PDCD4** | programmed cell death 4 |
| **PDGF** | platelet-derived growth factor |
| **PDX** | patient-derived xenograft |
| **PD-1** | programmed cell death protein 1 |
| **PD-L1** | programmed death-ligand 1 |
| **PEP** | phosphoenolpyruvate |
| **pEMT** | partial epithelial–mesenchymal transition |
| **PGE2** | prostaglandin E2 |
| **PI** | propidium iodide |
| **PI3K-AKT** | phosphoinositide 3-kinase–protein kinase B |
| **PlGF** | placental growth factor |
| **PRISMA** | Preferred Reporting Items for Systematic Reviews and Meta-Analyses |
| **PRR** | pattern recognition receptor |
| **PTEN** | phosphatase and tensin homolog |
| **PYD** | pyrin domain |
| **RAGE** | receptor for advanced glycation end-products |
| **RIG-I** | retinoic acid–inducible gene I |
| **RNS** | reactive nitrogen species |
| **ROS** | reactive oxygen species |
| **rRNA** | ribosomal RNA |
| **S100A8 / A9** | S100 calcium-binding proteins A8 / A9 (calprotectin) |
| **SCFA** | short-chain fatty acid |
| **snRNA** | small nuclear RNA |
| **SMAD** | small mothers against decapentaplegic |
| **SOCS** | suppressor of cytokine signalling |
| **SPM** | specialised pro-resolving mediator |
| **STAT** | signal transducer and activator of transcription |
| **sTNFR** | soluble TNF receptor |
| **sIL-6R** | soluble IL-6 receptor |
| **STING** | stimulator of interferon genes |
| **TAM** | tumour-associated macrophage |
| **TAM receptors** | Tyro3 / Axl / Mer receptor family |
| **TAN** | tumour-associated neutrophil |
| **TCA** | tricarboxylic acid (cycle) |
| **TCF / LEF** | T-cell factor / lymphoid enhancer factor |
| **TCR** | T-cell receptor |
| **TF** | tissue factor |
| **TGF-β** | transforming growth factor beta |
| **Th1 / Th17** | T helper 1 / 17 cell |
| **TIGIT** | T-cell immunoreceptor with Ig and ITIM domains |
| **TIM-3** | T-cell immunoglobulin and mucin domain–containing 3 |
| **TLR** | Toll-like receptor |
| **TMA** | trimethylamine |
| **TMAO** | trimethylamine N-oxide |
| **TME** | tumour microenvironment |
| **TNF-α** | tumour necrosis factor alpha |
| **TNFAIP3** | TNF-α–induced protein 3 (synonym A20) |
| **TNFR** | TNF receptor |
| **TNM** | tumour–node–metastasis (staging system) |
| **TRAF6** | TNF receptor–associated factor 6 |
| **TRAIL** | TNF-related apoptosis-inducing ligand |
| **Treg** | regulatory T cell |
| **TSP-1** | thrombospondin-1 |
| **VCAM-1** | vascular cell adhesion molecule 1 |
| **VE-cadherin** | vascular endothelial cadherin |
| **VEGF** | vascular endothelial growth factor |
| **VEGFR1 / 2** | vascular endothelial growth factor receptor 1 / 2 |
| **vWF** | von Willebrand factor |
| **YAP** | Yes-associated protein |
| **ZEB1** | zinc finger E-box binding homeobox 1 |
| **γH2AX** | γ-H2A histone family member X |
